# Time-lapse single-cell transcriptomics reveals modulation of histone H3 for dormancy breaking in fission yeast

**DOI:** 10.1038/s41467-020-15060-y

**Published:** 2020-03-09

**Authors:** Hayato Tsuyuzaki, Masahito Hosokawa, Koji Arikawa, Takuya Yoda, Naoyuki Okada, Haruko Takeyama, Masamitsu Sato

**Affiliations:** 1 Department of Life Science and Medical Bioscience, Graduate School of Advanced Science and Engineering, Waseda University, 2-2 Wakamatsucho, Shinjuku-ku, Tokyo 162-8480 Japan; 2 Computational Bio Big-Data Open Innovation Laboratory (CBBD-OIL), National Institute of Advanced Industrial Science and Technology, 3-4-1 Okubo, Shinjuku-ku, Tokyo 169-8555 Japan; 3 Research Organization for Nano and Life Innovation, Waseda University, 513 Waseda-tsurumaki-cho, Shinjuku-ku, Tokyo 162-0041 Japan; 4 Institute for Advanced Research of Biosystem Dynamics, Waseda Research Institute for Science and Engineering, Graduate School of Advanced Science and Engineering, Waseda University, 3-4-1 Okubo, Shinjuku-ku, Tokyo 169-8555 Japan; 5 Instituto de Biologia Molecular e Celular, Instituto de Investigação e Inovação em Saude (i3S), Universidade do Porto, 208 Rua Alfredo Allen, 4200-135 Porto, Portugal; 6 Institute for Medical-oriented Structural Biology, Waseda University, 2-2 Wakamatsucho, Shinjuku-ku, Tokyo 162-8480 Japan

**Keywords:** Gene expression analysis, Cell division, Computational biology and bioinformatics, Microbiology

## Abstract

How quiescent cells break dormancy is a key issue in eukaryotic cells including cancer. Fungal spores, for example, remain quiescent for long periods until nourished, although the mechanisms by which dormancy is broken remain enigmatic. Transcriptome analysis could provide a clue, but methods to synchronously germinate large numbers of spores are lacking, and thus it remains a challenge to analyse gene expression upon germination. Hence, we develop methods to assemble transcriptomes from individual, asynchronous spore cells of fission yeast undergoing germination to assess transcriptomic changes over time. The virtual time-lapse analyses highlights one of three copies of histone H3 genes whose transcription fluctuates during the initial stage of germination. Disruption of this temporal fluctuation causes defects in spore germination despite no visible defects in other stages of the life cycle. We conclude that modulation of histone H3 expression is a crucial ‘wake-up’ trigger at dormancy breaking.

## Abstract (web summary)

Asynchronicity in fungal spore germination makes transcriptomic analysis of the process challenging. Here, the authors assay single cell transcriptomes of germinating yeast cells and find that one of the histone H3 genes shows fluctuating expression, disruption of which causes germination defects.

## Introduction

**Fig. 1:**
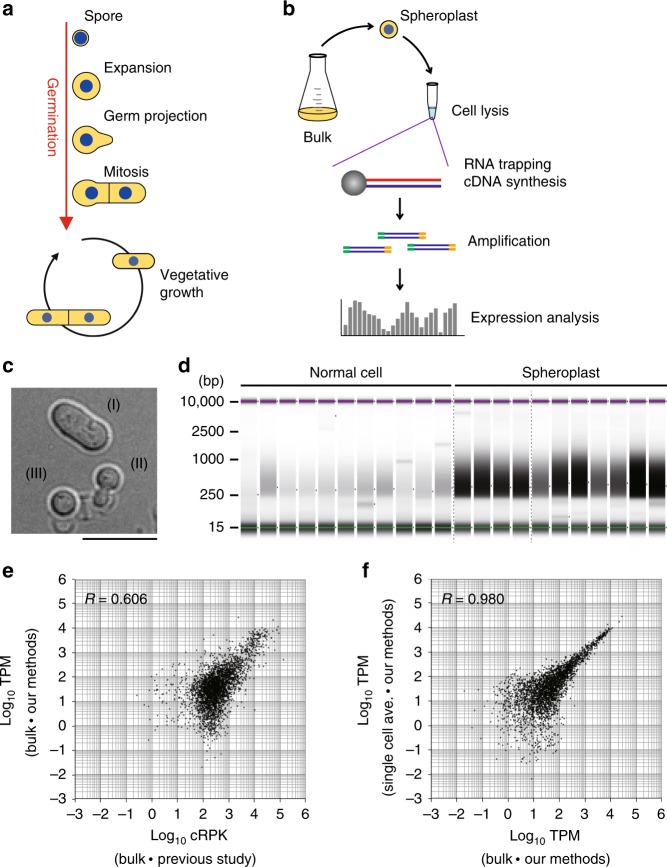
Validation of single-cell transcriptome methods. **a** Germination processes and entry into vegetative growth of *S. pombe* cells. **b** Overview of the bead-based scRNA-seq developed in this study. **c** Differential interference-contrast images of WT cells after treatment with lysing enzyme: (I) cells not lysed, (II) cells being lysed, and (III) cells that were substantially lysed. Representative cells of >10 independent experiments are shown. Scale bar, 10 µm. **d** Assessment of scRNA-seq library from 22 single cells for which spheroplasts were prepared or not prepared (Normal cell), as assessed with electrophoresis. Each lane corresponds to a sample from a single cell. Size (bp) is shown to the left. Data are representative of two independent experiments. Uncropped original image is shown in Supplementary Fig. 8. **e** Comparison of *S. pombe* transcriptomes created from bulk cells in this study and in a previous study^9^. **f** Comparison of single-cell-based transcriptomes (average of 11 cells) and transcriptomes created from bulk cells in this study. TPM transcripts per million mapped reads, cRPK reads per kilobase of length corrected for mappability. Source data are provided as a Source Data file.

Eukaryotic cells of fungi, plants and animals often exist in a non-dividing state called quiescence or dormancy. However, cells may begin to proliferate upon a change in environmental conditions such as nutrient availability or signalling from neighbouring cells. The fission yeast *Schizosaccharomyces pombe* undergoes meiosis and gametogenesis (sporulation) under nutrition starvation to generate round-shaped spores that remain dormant until nutrients become available (Fig. 1a)^1^. Nutrition refeeding gradually breaks spore dormancy, and germinating spores undergo morphological changes such as cell wall expansion accompanied by cytoskeletal rearrangements to form a germ projection (Fig. 1a)^2–4^. The cell cycle is then activated.

It is likely that these dormancy-breaking events are brought about by alterations in gene expression upon feeding, although the genes involved have not been fully elucidated. Therefore, defining the temporal changes in gene expression profiles would provide direct evidence concerning the mechanism of germination. The creation of transcriptional profiles for cells at germination requires that total cellular RNA be harvested from a large number of cells undergoing germination^5^. In budding yeast, synchronous germination can be achieved to some degree by selecting optimal nutrition conditions to build transcription profiles^6^. It appears, however, that in fission yeast individual cells tend to break dormancy at a distinct time, and no methods have been established to synchronously induce spore germination for bulk spore cells.

To overcome this technical limitation, we carry out single-cell transcriptome profiling (single-cell RNA sequencing (scRNA-seq)) to create transcriptional profiles from single cells at the state of our interest (such as a germinating spore). This approach bypasses the need for synchronised cultures. As there are few examples of single-cell-based genomic analyses in yeast^7,8^, we first determine whether we can faithfully reproduce the transcriptomes of vegetative cells in comparison with transcriptomes derived from bulk cultures using standard methods^9^. The *S. pombe* scRNA-seq methods are then expanded to single spore cells. In combination with bioinformatics, we draw a landscape how transcriptomes change over time during germination processes. Through the technical advance, we discover that histone H3 levels fluctuate in reaction to germination cues, event of which is essential to promote dormancy breaking.

## Results

### Establishing single-cell transcriptomes

The overall strategy is presented in Fig. 1b. First, we utilised spheroplasting of cells prepared in bulk culture to effect severe damage of the cell wall, as indicated by a change to a round cell shape (Fig. 1c). Each single cell was picked up and then chemically lysed to extract poly(A)+ RNAs, which were then captured with nanoparticle beads (see Fig. 1b), on which reverse transcription and subsequent procedures were performed so that double-stranded cDNAs could be prepared. We particularly applied the “bead-seq” method^10^ to minimise biased amplification of cDNAs and thereby reduce the background noise during data analysis. For a quality check, cDNA library preparation was confirmed through electrophoresis (Fig. 1d). The cDNA library pool was then deep sequenced and mapped on the reference genome to assemble a transcriptome (scRNA-seq).

To evaluate whether our methods generated high-quality transcriptomes, we compared our single-cell-based scRNA-seq transcriptome with a published profile created from bulk cells using conventional methods^9^. When the data for each of ~7000 *S. pombe* annotated genes was plotted versus the expression level measured in those two distinct transcriptomes, the correlation coefficient was *R* = 0.606, indicating that, on average, gene expression was detected at similar levels in the two transcriptomes, although there were certain genes that showed different expression levels (Fig. 1e). This may be attributable to “individuality” in the expression of genes in individual cells or to differences in protocols. It is also possible that the difference was arisen because our sequencing was not directional. A comparison of profiles created from individual cells and bulk (~10) cells using our same methods revealed only subtle differences (*R* = 0.980, Fig. 1f), indicating that any difference with published profiles (Fig. 1e) could be attributed to technical differences and that our scRNA-seq protocol for single-cell transcriptome assembly yielded results comparable with those from studies of bulk cells (Fig. 1f).

### Expanding the scope of the protocol to single spores

**Fig. 2:**
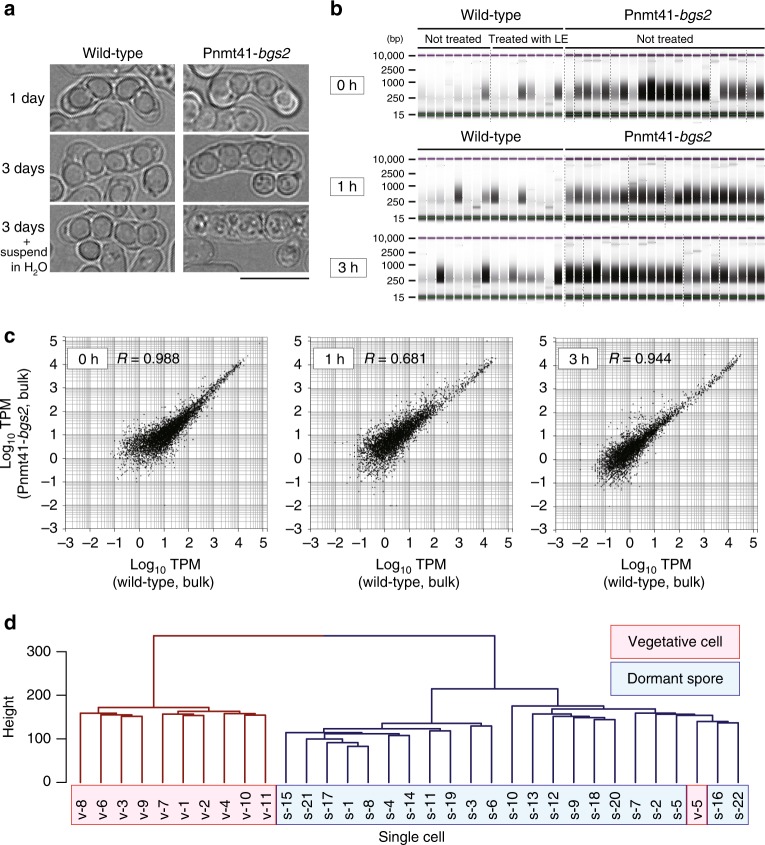
scRNA-seq-based transcriptome assembly from a single spore cell. Strain Pnmt41-*bgs2* was used to efficiently prepare cDNAs from dormant or germinating spores. **a** Strain Pnmt41-*bgs2* undergoes meiosis but produces fragile spores. WT (*bgs2*^+^) spores could tolerate water, whereas Pnmt41-*bgs2* spores burst. Representative cells of three independent experiments are displayed. Scale bar, 10 µm. **b**, **c** Spores of the indicated genotypes were picked at 0, 1 and 3 h after nutrition feeding. **b** cDNAs were not produced in sufficient amounts from WT cells irrespective of whether they were treated with the lysing enzyme (left), whereas cDNA could be produced in sufficient amounts from Pnmt41-*bgs2* spores even without lysing enzyme treatment (right). Uncropped original image is shown in Supplementary Fig. 8. **c** Global gene expression measured in strain Pnmt41-*bgs2* did not differ significantly from that of the WT cells. **d** Transcription profiles of single vegetative cells (v-1–v-11, red) and unfed dormant spores (s-1–s-22, blue) were classified into distinct groups through an informatic operation. Relative height reflects the similarity between transcriptomes. Source data are provided as a Source Data file.

Our protocol was then applied to create single-spore transcriptomes, but a sufficient amount of RNA could not be extracted from a single spore cell, probably because the hard spore wall hampered cell lysis (see below). To circumvent this issue, we engineered the Pnmt41-*bgs2* strain to reduce the expression of β-glucan synthase, which is required specifically for spore-wall synthesis but not for cell wall integrity during vegetative growth^11^. In standard medium, the Pnmt41-*bgs2* strain produced spores with a fragile spore wall (Fig. 2a), and indeed the spores burst when suspended in water (Fig. 2a). Thus spore lysis could be achieved without using lysing enzymes, which is a great technical advantage for enabling single-spore omics.

Wild-type and Pnmt41-*bgs2* spores were fed with nutrients to induce germination, and single spores were picked at 0, 1 and 3 h and then burst in water. RNA-seq library was more efficiently amplified from single cells of Pnmt41-*bgs2* spores than from wild-type (WT) spores (Fig. 2b). In addition, the reduction in Bgs2 expression in Pnmt41-*bgs2* did not affect the overall transcription profile in dormant or germinating spores (Fig. 2c and Supplementary Fig. 1). Thus the improved method was validated for profiling gene expression in a single spore.

Having established experimental procedures, the transcriptomes of single spores were compared to those of single vegetative cells (Fig. 2d). A total of 33 single-cell transcriptomes from vegetative cells and unfed dormant spores were sorted out into two major groups. One group exclusively comprised vegetative profiles, and the other comprises profiles mostly from dormant spores (Fig. 2d), verifying that our single-cell methodology is sensitive enough to detect changes in transcription in cells exposed to different nutrient conditions.

### Estimating temporal changes in the gene expression

To specifically focus on genes for which expression varied during the initial stage of germination, it was necessary to determine which transcriptome was derived from an individual spore at that stage. This was impossible to deduce based on the appearance of spores under a wide-field microscope used when they were picked through manipulation of a micropipette: spores at the initial stage appeared morphologically similar there. For that purpose, we collected 64 spores at 0, 1 and 3 h after induction of germination by nutrition refeeding (20–22 spores from each time point) and assembled transcriptomes for each spore. To determine which transcriptomes corresponded to spores that were at the initial stage of germination, we implemented the “monocle” operation (see below) to compare differences in all 64 transcriptomes, thereby deducing the temporal shift in transcriptomes from one to another^12,13^. Variation between any two transcriptomes implies a temporal shift from one to another, and therefore any small variation could be derived from two successive transcriptomes over time. Repeating the monocle operation thus enables the clustering of all transcriptomes, from which changes in transcription profiles can be estimated over time, facilitating the temporal alignment of profiles to produce a virtual timeline.

**Fig. 3:**
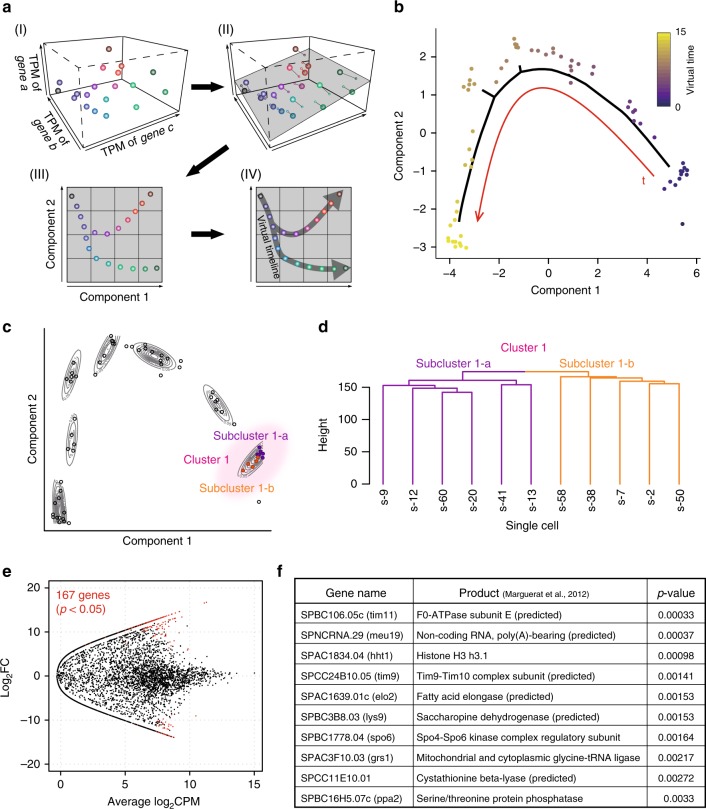
Transcriptomes could be aligned along a virtual timeline. Bioinformatics approaches were applied to estimate temporal changes in gene expression profiles during germination. **a** Schematics illustrating the idea of the monocle-based similarity judgement. (I) Assuming that a cell contains three genes (a–c). In this example, transcriptomes can be plotted in three-dimensional space (dots in the graph) depending on the extent to which these genes were expressed. (II) Those data points can be projected onto a single plane (grey) so that the number of dimensions can be reduced from 3 to 2, as depicted in (III). In the two-dimensional plane, the Euclidean distance between data points indicates the similarity between two transcriptomes, and this similarity is used to estimate the time flow of the virtual timeline through a minimum spanning tree (IV). **b** Two-dimensional transcriptome plots, which were compressed from ~7000 dimensions using repetitive monocle operations. We used 64 samples created from single spores before and after nutrition feeding (0, 1 and 3 h). The red curve indicates the virtual timeline, and transcriptomes were aligned in the time course indicated by the heat map. **c** Plots in **b** were classified into seven clusters along the virtual timeline. Cluster 1 (pink) corresponds to the very initial stage of germination estimated from the timeline, and plots therein could be further divided into two subclusters (1-a, purple; and 1-b, orange). **d** Transcriptomes in two subclusters were divided into distinct groups through an informatic operation. **e** The expression of ~7000 annotated genes (dots) was compared between subclusters 1-a and 1-b. An exact test (two-sided, *p* < 0.05) revealed 167 genes for which expression varied significantly between these subclusters (shown in red). **f** The top 10 genes with the highest *p* values among the 167 variable genes. Gene products were inferred from Marguerat et al^9^. Source data are provided as a Source Data file.

Figure 3a illustrates how the monocle operates: we applied the monocle operation to an imaginary transcriptome comprising expression data for three genes, namely “a”, “b”, and “c”, in terms of transcripts per million mapped reads (TPM) (Fig. 3a-I). In this case, each transcriptome can be plotted in three-dimensional space depending on the expression of each gene. The monocle operation projects the three-dimensional location of each transcriptome onto a two-dimensional plane (Fig. 3a-II, a-III). As spatial distance on the two-dimensional plane reflects temporal distance, the monocle operation estimates the timeline along with spatial proximity data (Fig. 3a-IV).

The *S. pombe* transcriptome comprises a data set of ~7000 dimensions corresponding to expression levels (i.e., TPM) of ~7000 annotated genes. By reducing the dimension from ~7000 down to 2 in a stepwise manner, the data for all 64 samples could be plotted on a two-dimensional map, and the virtual timeline was drawn by monocle operations connecting proximal plots one by one (Fig. 3b). The plotted transcriptomes were divided into seven major clusters along the virtual timeline, with the first cluster (Cluster 1, Fig. 3c) corresponding to spores at the initial stage of germination.

We identified genes for which expression varied significantly from one cluster to another (e.g. from Cluster 1 to 2) using gene ontology (GO slim) terms (Supplementary Fig. 2). Proteins involved in the metabolism appeared variable throughout germination processes, but in later stages (Cluster 6 to 7), factors for chromatin organisation were found variable, which may suggest that global chromatin remodelling occurs right before entry into the first cell cycle.

When the cluster analysis was applied to transcriptomes in Cluster 1, it was divided into subclusters 1-a and 1-b (Fig. 3c, d). We focused on genes for which expression varied significantly between these two subclusters, and 167 genes revealed significant variation in expression (*p* < 0.05, Fig. 3e). Genes with GO slim terms “mRNA metabolic process”, “lipid metabolic process”, “nucleotide-containing small molecule metabolic process”, “ascospore formation” and “detoxification” were particularly enriched in the 167 variable genes (Supplementary Fig. 3). The top ten most variable genes are listed in Fig. 3f.

### Histone H3 fluctuates for germination

**Fig. 4:**
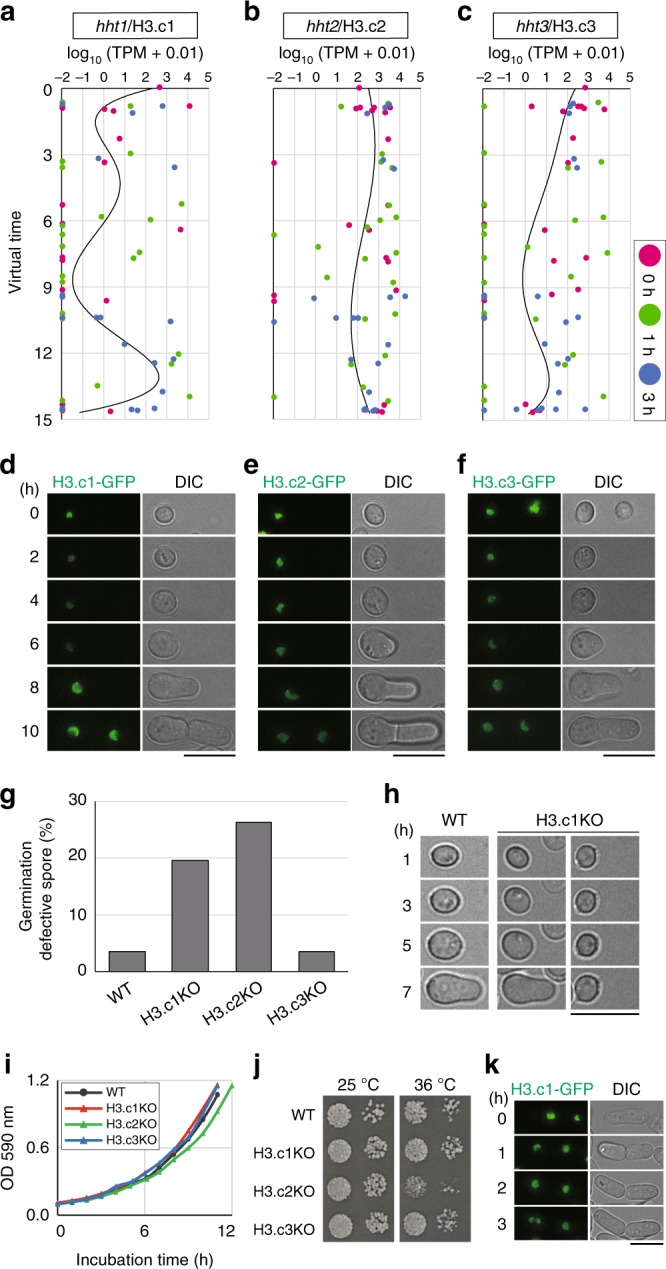
Expression of the histone H3 gene H3.c1 fluctuates during germination. The expression of one of the identified genes, *hht1* (H3.c1), indeed fluctuates. **a**–**c** *S. pombe* has three histone H3 genes: *hht1*/H3.c1 (**a**), hht2/H3.c2 (**b**), and *hht3*/H3.c3 (**c**). Expression of each histone H3 gene detected in 64 transcriptomes shown in Fig. 3 was plotted versus the virtual timeline. Magenta, 0 h; green, 1 h; blue 3 h after feeding. **d**–**f** Each H3 protein fused with GFP was expressed under the native promoter at the endogenous level. Live-cell imaging started from dormant spores (0 h) until cells finished the first division. The fluorescence of only H3.c1-GFP fluctuated significantly (**d**). Representative of >10 independent cells are shown. Scale bar, 10 µm. **g**, **h** Knockout of H3.c1 (H3.c1KO) or H3.c2 causes defects in germination. **g** Frequencies of cells with defective germination in WT and knock-out strains of each H3 gene. *n* > 50 spores for each strain, *N* = 2 independent experiments. Values averaged in the graph are given below. WT, 2.3% and 4.7%; H3.c1KO, 23.4% and 15.8%; H3.c2KO, 28.2% and 24.4%; H3.c3KO, 4.0% and 2.9%. **h** Shown are live-cell differential interference-contrast (DIC) images of WT and H3.c1KO spores at germination. Hours (h) indicate time after filming started. Scale bar, 10 µm. **i**, **j** H3.c1KO showed no visible growth defects during vegetative cycles, whereas H3.c2KO showed slight defects. H3.c2KO showed slight growth defects at 25 °C (**i**), which were even more evident on the plate at 36 °C (**j**). Black circles, WT; red triangles, H3.c1KO; green triangles, H3.c2KO; blue triangles, H3.c3KO. Growth curves were drawn with 3 × 10^5^ cells at 0 h. Uncropped original image is shown in Supplementary Fig. 8. **k** The level of H3.c1-GFP did not fluctuate during vegetative cycles in single-cell time-lapse observations. Data are representative of >10 independent cells. Scale bar, 10 µm. Source data are provided as a Source Data file.

Within the gene list of Fig. 3f, we particularly focused on the gene *hht1* (encoding histone H3, copy 1; hereafter H3.c1); notably, *S. pombe* has three histone H3 genes (H3.c1, H3.c2, H3.c3), but it is unclear whether these genes are differentially used in *S. pombe*, as all three copies encode identical amino acid sequences^14^. A monocle-based screen revealed that *hht1*/H3.c1 expression significantly varied upon germination, whereas the expression of *hht2*/H3.c2 or *hht3*/H3.c3 did not change after germination. Variation of three H3 transcripts along the virtual timeline after refeeding demonstrated that the level of the H3.c1 mRNA decreased immediately after refeeding but later increased, probably reflecting the timing of DNA replication, whereas that of H3.c2 and H3.c3 remained essentially constant (Fig. 4a–c). When the 167 variable genes were categorised with regard to similarity in temporal expression patterns, 9 other genes were found to display similar expression patterns to H3.c1, 4 of which have been GO termed as “nucleobase-containing small molecule metabolic process” (Supplementary Fig. 4).

These H3 expression patterns are reminiscent of those previously reported for the H3.c1 and H3.c3 transcripts in proliferating cells, which showed a peak at S phase, whereas H3.c2 stayed constant^15^. The expression level of H3.c1 mostly represents sense transcripts, as antisense transcripts of the H3.c1 gene was ~1% of sense transcripts^16^.

To monitor protein levels of histone H3, a knock-in of the gene encoding green fluorescent protein (GFP) was carried out in *S. pombe*, resulting in the expression of a *GFP* fusion with each of the genes encoding H3.c1, 2 and 3 under control of their native promoters (Fig. 4d–f). H3.c1-GFP fluorescence disappeared after germination was induced, but the fluorescence recovered to a considerable level at 8 h, when germ projection was evident (Fig. 4d). In contrast to the large fluctuations in the H3.c1-GFP signal, H3.c2-GFP fluorescence remained constant during germination, and H3.c3-GFP fluorescence fluctuated to a lesser extent than did that of H3.c1-GFP (Fig. 4e, f). Indeed, time-lapse behaviour of all H3 proteins as well as many of other top 10 proteins was consistent with the kinetics predicted for each of the H3 mRNAs (Fig. 4a–c; Supplementary Fig. 5), validating our estimation of the virtual timeline.

We then tested whether the three H3 proteins contributed to spore germination. Both H3.c1 knock-out (H3.c1KO) and H3.c2KO spores exhibited defective germination, as indicated by a significant delay or stall in the formation of the germ projection, whereas H3.c3KO spores germinated almost normally (Fig. 4g, h). In contrast to the germination defects, H3.c1KO cells did not exhibit visible growth defects during mitotic cell cycle, whereas H3.c2KO showed temperature sensitivity and slight growth defects during the mitotic cycle (Fig. 4i, j)^15^. In line with these results, the fluctuation in H3.c1-GFP fluorescence seen during germination was not evident in the mitotic cycle (Fig. 4k), demonstrating that H3.c1 expression is modulated specifically during germination. We thus postulated that H3.c1 is not just a redundant histone H3 but rather a variant that plays a role specifically in promoting germination, whereas H3.c2 is a general and housekeeping histone expressed and required at all time.

The germination defects seen in H3.c1KO spores might have arisen during meiosis or sporulation in parental zygotes, rather than defects in germination per se. Chromosome segregation pattern during meiosis, however, was almost normal in H3.c1KO zygotes (Supplementary Fig. 6a), excluding the possibility of aneuploidy of H3.c1KO spores. To further exclude the possibility of meiotic defects, if any, both H3.c1^+^ and H3.c1KO spores were prepared as “littermates” to equalise conditions of meiosis and sporulation by using the same parental cells (Supplementary Fig. 6b). As H3.c1KO spores showed significantly higher rate of defective germination than the H3.c1^+^ littermate control (Supplementary Fig. 6c), we conclude that defective germination in H3.c1KO spores was not originated from meiosis of parental cells. We also examined whether H3.c1KO cells were defective in maintenance of the spore integrity during dormancy. H3.c1KO spores showed defects in germination after 3-day dormancy, but the increase rates of the defects during the longer period of dormancy were indistinguishable in H3.c1KO and H3.c1^+^ spores (Supplementary Fig. 6d). This result suggests that the high rate of defective germination in H3.c1KO in comparison with H3.c1^+^ cells are not due to defects in spore maintenance.

### Modulation of histone H3 expression during germination

**Fig. 5:**
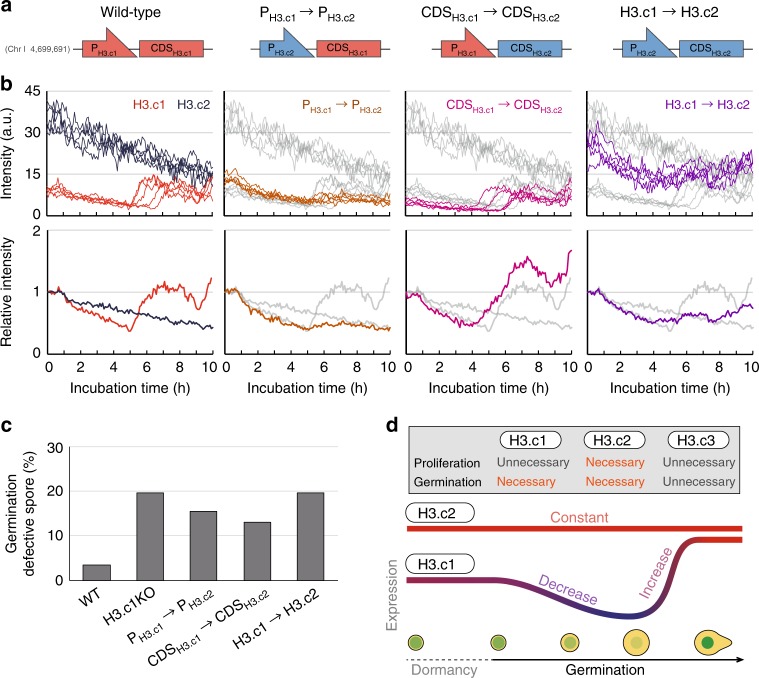
Control of H3.c1 expression by its promoter is key for fluctuation. Temporal changes in the levels of the various histone H3 proteins are controlled by their promoters (P) and coding sequences (CDS). **a** Schematics illustrating states of H3.c1 and H3.c2 gene loci used in this study. For example, P_H3.c1_ → P_H3.c2_: the strain in which the promoter of H3.c1 (P_H3.c1_) was replaced with that of H3.c2 (P_H3.c2_); H3.c1 → H3.c2: both P and CDS were exchanged. **b** Fluorescence intensity of GFP was measured over time. Top, absolute values are shown; bottom, intensities were normalised to values at time 0 (start of observation). Intensities of WT H3.c1-GFP (red) and H3.c2-GFP (dark blue) are shown to the left, which are also copied onto other graphs (grey traces). Similarly, P_H3.c1_ → P_H3.c2_-GFP (brown), CDS_H3.c1_ → CDS_H3.c2_-GFP (magenta), and H3.c1 → H3.c2-GFP (purple) are shown. **c** Artificial alteration of H3.c1 expression caused defects in germination. *n* > 70 spores, *N* = 2 independent experiments. Values averaged in the graph are given below. WT, 2.3% and 4.7%; H3.c1KO, 23.4% and 15.8%; P_H3.c1_ → P_H3.c2_, 11.6% and 19.3%; CDS_H3.c1_ → CDS_H3.c2_, 10.8% and 15.2%; H3.c1 → H3.c2, 18.9% and 20.3%. **d** A model for histone H3.c1 expression for efficient germination. Expression decreases once in the initial stage but is substantially boosted in the later stages of germination. Source data are provided as a Source Data file.

Because the amino acid sequences of the three H3.c1–3 proteins are identical, we speculated that the observed differential expression of H3.c1 and H3.c2 was a consequence of differences in their DNA sequences in the 5’ promoter or coding region. The 3’ terminator region cannot account for the difference because each of the engineered fusion genes (H3.c1-GFP, etc.) contained the same exogenous terminator (T_ADH_), and indeed their expression patterns were unaffected by this (see Fig. 4d). We therefore replaced the promoter for H3.c1 (P_H3.c1_) with the one for H3.c2 (P_H3.c2_) at the endogenous locus (the strain “P_H3.c1_ → P_H3.c2_”, Fig. 5a) to evaluate the effect of P_H3.c1_ on the kinetics of H3.c1 expression. After inducing germination in WT cells, the level of H3.c1-GFP gradually decreased but then increased significantly after 5 h (Figs. 4d–f and 5a, b; Supplementary Fig. 7). In contrast, this increase was not observed when P_H3.c1_ was replaced with P_H3.c2_ (P_H3.c1_ → P_H3.c2_, Fig. 5a, b), indicating that the promoter region of H3.c1 is responsible for the observed transcriptional boost of H3.c1 during germination.

Strain P_H3.c1_ → P_H3.c2_ displayed defects in spore germination, indicating that the observed transcriptional boost ensured that germination would progress (Fig. 5c). Replacement of the coding sequence (CDS) of H3.c1 with that of H3.c2 (“CDS_H3.c1_ → CDS_H3.c2_”, Fig. 5a) reduced the expression even at the very initial stage of germination, which might account for the minor defects in germination observed for this mutant, as the boost at later stages occurred normally (Fig. 5b, c). Replacement of both the promoter and coding regions of H3.c1 by those of H3.c2 (“H3.c1 → H3.c2”, Fig. 5a) resulted in its constitutive expression, which was comparable with the expression kinetics of endogenous H3.c2 (Fig. 5b). This implied that the total H3 level in this strain was consistently high even from the initiation stage of germination. This strain had a high rate of defective germination (Fig. 5c), demonstrating that the cells needed to repress the H3 expression to avoid an overabundance early during germination, whereas there was need to boost expression at a later stage around DNA replication. Thus spores emerge from dormancy via temporal upregulation of a specific H3 gene using the promoter and coding regions of that H3 gene, even though all three H3 proteins have identical amino acid sequences (Fig. 5d).

## Discussion

We took the approach of single-cell-based transcriptomes to investigate the long-lasting issue of how the dormancy of spores is broken in response to environmental change. Only a single example of *S. pombe* single-cell transcriptomics has been published^8^; by comparison, our method entails easier handling procedures and requires no specialised devices, and indeed it yields a higher rate of gene detection than the previous protocol. Statistical comparison of gene expression profiles created from a single cell and from bulk cells demonstrated that the validity of the single-cell protocol matches that of bulk cells (see Fig. 1e, f). As a proof of principle, we utilised bioinformatics to align transcriptomes from single cells at various stages of germination along a virtual timeline. This analysis highlighted *hht1*, one of three histone H3 genes in *S. pombe*, as a candidate gene for which expression is altered at the initial stage of germination (see Fig. 3). Analysis of the temporal kinetics of mRNA levels estimated along the virtual timeline revealed a correlation with the fluorescence emitted by histone H3-GFP proteins as assessed with live-cell imaging, further validating our methodology (Fig. 4a–f). Based on the expression data acquired over time (Fig. 3c), the 64 transcriptomes we created from single cells comprised 7 clusters that reflected the various stages of germination. Indeed, each stage may reflect the progress of stepwise cellular events, e.g. morphological alterations and DNA replication prior to entry into proliferation cycles.

A technical concern might be that single-cell pick-up could cause stress responses that affect gene expression. The risk, however, has been minimised by the following tips: (1) for vegetative cells: immediate freezing of a single cell right after pick-up. (2) For spore cells, use of the Pnmt41-*bgs2* mutant allowed an immediate burst of a spore after soaking in the solution.

Our study demonstrates functional differences between the three copies of histone H3 genes encoding identical amino acid sequences. Hht1/H3.c1 and Hht2/H3.c2 play major roles in germination, whereas Hht3/H3.c3 is dispensable. Previous studies revealed that expression of both H3.c1 and H3.c2 in proliferating cells peaked in S phase^15^. We, however, found that the H3.c1 level decreased in the initial stage of germination and then was upregulated in later stages, whereas H3.c2 was continuously expressed at a high level (Fig. 5a, b), indicating that their expression during germination is regulated in a manner distinct from that of mitotic cycles. In previous transcriptome analyses using cells that were shifted from G0 quiescence to the cycling stage, no H3 genes were found to have altered transcription^17^, suggesting that changes in H3.c1 expression is specific in spore germination. We envision that, at the end of gametogenesis, spores fix their chromatin a state to ensure survival during prolonged dormancy, and this state must be reset upon germination via the downregulation of H3.c1 expression, which allows yeast cells to establish a nutrient-replete cellular programme and proliferate.

We propose that cooperative expression of H3.c2 (continuous expression) and H3.c1 (variable) is essential for efficient germination (Fig. 5d). In this regard, within dormant spores, histone H3 proteins (H3.c1 and H3.c2) are abundant in nucleosomes, but some population of histone H3 must be downregulated at the initial stage of germination in response to changes in nutrient availability, which may contribute to a relaxation of chromatin structure to enable a global transcriptional boost. Two histone H3 genes show distinct properties in expression: the H3.c1 gene is controllable in reaction to environmental cues, whereas the H3.c2 gene remains constitutive, possibly serving as a pool of housekeeping histones for nucleosome assembly. Thus flexibility and robustness of histone H3 expression may be achieved by combinatory expression of two differential histone H3 genes.

The expression of histone H3 genes might be altered depending upon the protein level of histone on its own. However, two H3 genes were differentially expressed upon germination, although there is only one type of H3 protein (H3.c1 = H3.c2). This implies that expression of H3 genes is regulated by germination cues rather than simple autoregulation by the total H3 level (=H3.c1 + H3.c2), although we cannot exclude the possibility that H3 autoregulates only H3.c1 expression, but not H3.c2.

As germination proceeds, histone H3 level must be increased to associate with newly synthesised DNA during S phase of the pioneer round of the cell cycle. For that purpose, the controllable H3.c1 gene can be utilised again. The transcriptional boost can be ascribed to the promoter activity of the H3.c1 gene (Fig. 5a, b). In contrast, the H3.c1 CDS appears to be required for maintenance of its expression to an optimal level from the very initial stage of germination, possibly because transcripts that contain the H3.c1 CDS might be more stable than those that contain the H3.c2 CDS at that time. Alternatively, the chromatin region that includes the H3.c1 locus may be silenced (e.g. facultative heterochromatin), and the H3.c1 CDS might open the chromatin to facilitate transcription of the H3.c1 gene at the later stage of germination. Concomitantly, in later stages of germination, genes involved in chromatin organisation (termed by GO slim; Cluster 6 to 7, Supplementary Fig. 2) may be activated, to further induce global gene expression.

At early points in the development of mammalian embryos, the genome-wide chromatin state (i.e. open or closed) may change dramatically to promote cell division^18,19^. The fission yeast is a simple and unique organism with respect to H3 structure, as all three copies are composed of identical amino acid sequences that are a hybrid of the canonical mammalian H3.2 and H3.3 sequences, which is unlike higher eukaryotes that have three distinct H3 sequences^20^. Hence, the evolutionarily simple fission yeast uses its three H3s in different ways by modulating the temporal kinetics of transcription of their individual genes to adapt to environmental changes and enter a routine mitotic state.

## Methods

### Yeast strains, media and genetics

Standard materials and methods were used for *S. pombe* biology^21^. Strains used in this study are listed in Supplementary Table 1. Briefly, YE5S (yeast extract with supplements) was used for vegetative growth and also as refeeding medium to induce the germination of spores. Edinburgh minimal medium supplemented with nitrogen sources and amino acids was used for the preparation of vegetative cells for RNA sequencing. Sporulation agar plates were used for induction of mating and meiosis.

The prototrophic strain (L975: h90 WT) was mainly used as a host strain for genome editing and also as a control strain for assessing germination, because background mutations such as those for leucine or uracil autotrophy can sometimes affect germination.

Conventional methods were used for gene knock-out and knock-in^22–24^. The sequences of the oligonucleotides used are listed in Supplementary Table 2. In knock-out strains, the CDS of interest was replaced with *hph* (Hygromycin B resistance gene). In GFP-tagged strains, *GFP* was knocked in at the 3’-end of the gene of interest. For instance, in strain hht1(H3.c1)-GFP, *GFP* was inserted at the 3’-end of the CDS of *hht1* (in frame) to replace the endogenous *hht1* with the *hht1*-GFP fusion gene. This ensured that the expression of *hht1-GFP* remained under control of the native *hht1* promoter. Strain Pnmt41-*bgs2* was created using the template plasmid pFA6a-bsdMX6-pnmt41, in which the kanMX6 of pFA6a-kanMX6-pnmt41 was replaced with bsdMX6. The Pnmt41 promoter is a thiamine-repressible promoter, which moderately induces expression of the downstream gene in medium lacking thiamine^25^.

For vegetative growth assays, cells were grown in YE5S liquid medium at 25 °C overnight until 10^6^–10^7^ cells per ml was reached (“precultures”). For liquid culture–based assays, the preculture was inoculated into 10 ml YE5S medium, which was then placed in a shaker incubator (130 rpm) at 25 °C overnight. OD_590_ values were measured every hour using a spectrophotometer (GeneQuant 1300; GE Healthcare Life Sciences). For plate-based growth assays, the preculture was concentrated through mild centrifugation to 2 × 10^8^ cells per ml, after which 10-fold serial dilutions were made. Approximately 10^1^–10^6^ cells were spotted onto YE5S plates followed by incubation at 25 or 36 °C.

### Preparing spheroplasts

Fission yeast cells cultured in appropriate media were collected and washed once with Buffer A (1.2 M d-sorbitol, 50 mM citrate-phosphate, pH 5.6). Here we used cells expressing Ste11-GFP to pick up non-starved cells^26^. Cells were then suspended in 200 µl Buffer A containing 4 mg lysing enzyme from *Trichoderma harzianum* (Novozymes) and incubated at 32 °C for 30 min with stirring every 10 min. Cells were then washed three times with Buffer B (1.2 M d-sorbitol, 30 mM Tris-HCl, pH 7.6) and finally suspended with 50 µl Buffer B.

### Preparing spores and induction of germination

Pnmt41-*bgs2* mutant cells (for main experiments) as well as WT (expressing Ste11-GFP) were used for preparation of spores. Briefly, sexual differentiation (mating, meiosis and sporulation) was induced on solid sporulation agar plates without nitrogen sources for 3 days at 30 °C until spores were spontaneously separated. For induction of spore germination, cells containing spores were washed once with Buffer B and suspended into rich medium (i.e., YE5S) for 0–3 h (feeding) at 30 °C, unless otherwise stated. To pick up single spores, cells at each time point were collected and washed with Buffer B and then resuspended into 500 µl Buffer B prior to pick-up.

Littermate experiment (Supplementary Fig. 6) was conducted as follows. To equalise the conditions of meiosis when H3.c1^+^ (WT) and H3.c1KO spores are produced, parental H3.c1^+^ (HT0777) and H3.c1KO (HT0174) cells were conjugated and zygotic meiosis was induced. One of the parental strains (H3.c1^+^; HT0777) has been engineered in two respects: (1) the H3.c1^+^ gene has been marked by the GFP gene fused with *pmo25* gene, which is located close (~5 kb) to the H3.c1 gene. Namely, the H3.c1^+^ allele was marked by the Pmo25-GFP gene, whereas H3.c1KO allele was not. (2) An additional H3.c1^+^ gene has been introduced to a parental strain (HT0777) in order to compensate the lack of a copy of the H3.c1^+^ gene during meiosis of the heterozygous diploid H3.c1^+^/H3.c1KO. The additional copy of the H3.c1^+^ gene including the native promoter and terminator was inserted to a gene-free region near the *zfs1-mcherry* locus (at an ~5 kb distance; referred to as “Zfs1-mCherry::H3.c1”) of the strain. After the heterozygous diploid underwent meiosis and sporulation, we particularly focused on germination of littermate spores showing GFP(+) mCherry(–) (meaning H3.c1^+^ without the additional H3.c1^+^ gene) and GFP(–) mCherry(–) (H3.c1KO without the additional gene).

### Single-cell pick-up

Methods for the entire procedure are as follows. First, a bulk culture of cells was prepared in Edinburgh minimal medium (liquid) or on sporulation agar plates depending on experimental needs. Cells were collected and spheroplasts produced if necessary. Each cell suspension prepared in the previous step was mounted on a 35-mm dish and placed on the stage of a light microscope. With visual inspection through the eyepiece lenses, a Drummond Microdispenser (Drummond Scientific Company) pipette was used so that a single cell could be absorbed with 1 µl of buffer. Using the equipment, it was impossible to deduce the germination stage of individual spore cells, particularly those at the initial stage of germination. Each single cell (and spore cell) was randomly chosen and then transferred into a 0.2-ml tube containing 2.5 µl Collection Solution (2 U µl^−1^ RNase inhibitor and 0.2% (w/v) NP-40 in RNase-free ddH_2_O). Thus each sample was expected to contain a single cell in 3.5 µl liquid in total. Once collection was done, samples were stored at −80 °C.

### RNA extraction and cDNA preparation

For preparation of sequence libraries, samples were used for cDNA preparation with the “bead-seq” method^10^. Single-cell samples were defrosted, and 6.5 µl lysis solution was added; the final concentrations of reagents were as follows: 0.45× PCR buffer II (Life Technologies), 0.45% (w/v) NP-40, 4.5 mM dithiothreitol, 0.18 U µl^−1^ SUPERase•In (Ambion), 0.36 U µl^−1^ RNase inhibitor (Ambion), 0.15 mM each dNTP, dual-biotinylated anchored “oligo 1” primers immobilised on ~10^7^ beads per sample^10^. The sequences of the oligonucleotides used are listed in the Supplementary Table 2. Samples were transferred into a Caliper Zephyr Compact Liquid Handler (Perkin Elmer, CA, USA) coupled with a homemade operation programme based on the bead-seq method. Samples were then incubated by 70 °C for 5 min and chilled to room temperature, after which 5 µl reverse transcription solution was added (final concentration: 0.45× PCR buffer II, 1.35 mM MgCl_2_, 8 U µl^−1^ Superscript III reverse transcriptase from Life Technologies and 0.4 U µl^−1^ RNase inhibitor). Samples were incubated at 55 °C for 30 min followed by 70 °C for 5 min. Beads were magnetically collected in tube and washed with T&T buffer (0.1% (w/v) Tween 20, 10 mM Tris-HCl pH 8.0) and resuspended in 12 µl T&T buffer.

After reverse transcription, 6 µl of tailing solution (0.5× PCR buffer II, 0.75 mM MgCl_2_, 1.5 mM dATP, 0.15 U µl^−1^ RNase H, 0.188 U µl^−1^ terminal deoxynucleotidyl transferase) was added to the samples, with subsequent incubation in a shaker at 37 °C for 15 min followed by 70 °C for 10 min. Samples were then washed with T&T buffer once and resuspended with 12 µl T&T buffer. For synthesis of the second strand, 19 µl of each reaction mixture (final concentration: 1× Ex Taq buffer (TaKaRa Bio), 0.12 mM dNTP, 0.49 µM anchored “oligo 2” primers, 0.05 U µl^−1^ Ex Taq Hot Start version (TaKaRa Bio)) was added, and the samples were applied to a thermal cycler: 95 °C for 30 s, 44 °C for 5 min, and 72 °C for 6 min, followed by cooling to 4 °C.

Synthesised cDNA was then amplified via two-step PCR. For the first step, 1 µl of amplification solution 1 (1× Ex Taq buffer, 0.25 mM dNTP, 0.05 U µl^−1^ Ex Taq Hot Start version, 0.49 µM anchored “oligo1_T15” primer) was added to each sample, and thermal cycling was performed as follows: boiling at 95 °C for 0.5 min, followed by 18 cycles of 95 °C for 0.5 min, 67 °C for 1 min and 72 °C for 6 min, followed by cooling to 4 °C. Samples were then washed twice with 0.6 × AMPure XP reagent (Beckman Coulter) and resuspended with 20 µl T&T buffer.

For the second PCR step, 5 µl of each sample from the first PCR step was mixed with 45 µl of amplification solution 2 (1× Ex Taq buffer, 0.25 mM dNTP, 0.05 U µl^−1^ Ex Taq Hot Start version, 1 µM anchored “oligo1_T15” primer, 1 µM anchored “oligo 2” primers), and thermal cycling was performed as follows: boiling at 95 °C for 0.5 min, and 12 cycles of 95 °C for 0.5 min, 67 °C for 1 min and 72 °C for 6 min, followed by cooling to 4 °C. Samples were then washed once with 0.6 × AMPure XP reagent (Beckman Coulter) and resuspended with 20 µl T&T buffer.

### Sequencing

Amplified cDNA products were then fragmented for next-generation sequencing using the Nextera XT DNA Sample Prep Kit (Illumina). The resultant sequencing pool was subjected to TapeStation 4200 electrophoresis using High Sensitivity D5000 ScreenTape (Agilent Technology). Next-generation sequencing was then performed with HiSeq 2500 (Illumina; performed by Macrogen Corp., Japan). Pair-end reads of 101 nucleotides were sequenced.

### Computational analysis for gene expression levels

Read counts were calculated with RSEM-1.3.0^27^ and normalised to scores of TPM^28^. Read-out sequences in FASTQ-format files were mapped with RSEM-1.3.0: sequence reads containing up to 3 mismatches were mapped onto the reference genome of *S. pombe*: Schizosaccharomyces_pombe.ASM294v2.25.

### Estimation for the virtual timeline

For the drawing of scatter plots (Figs. 1e, f and 2c), the software Subio Platform ver.1.19 (Subio Inc., Amami, Japan) was used.

For virtual timeline ordering analysis (Fig. 3b), we utilised the R package monocle ver.2.4.0^12,13,29^. Matrices for TPM, cell attributes and gene annotation were used as input data; a TPM matrix was defined as a matrix for TPM values in which each row corresponds to each gene and each column to each single cell. A cell attribute matrix was defined as values for experimental conditions such as time after feeding (time for germination induction). Each row corresponds to a single cell and each column to a particular condition. A gene annotation matrix was defined as a gene information data set in which each row corresponds to a single gene and each column to annotated information. Input data were used for dimensionality reduction and virtual timeline deduction with reverse graph embedding methods^13^. For the monocle operation, standard parameters were used according to the instructions of the supplier, and genes with little or no expression (<0.1 RNA copies per cell) and genes detected in ≤10 samples were excluded from the operation.

For clustering of transcriptome data sets (Fig. 3c), the R package mclust ver.5.3^30,31^ was used. The output data set of the aforementioned monocle operation was used for mclust input. Samples were classified through Gaussian kernel density estimation. For further clustering using dendrograms (Fig. 3d), the R package amap ver.0.8.14 (https://CRAN.R-project.org/package=amap), and dendextend ver. 1.8.0^32^ were used to detect two subclusters 1-a and 1-b. The TPM matrices belonging to transcriptomes in Cluster 1, as classified by mclust, were used for input.

Similarity between samples (Figs. 2d and 3d) was estimated by measuring the Euclidean distance followed by hierarchical clustering with Ward’s method. Based on the similarity, two transcriptomes with the closest gene expression profiles were connected by a branch, the size of which is denoted as “height”. A pair of samples with a relatively low height value could be interpreted as having greater similarity.

For differential expression analysis as shown in Fig. 3e, the R package edgeR ver. 3.18.1^33,34^ was used. The read count matrices belonging to Cluster 1, as classified by mclust, were used for input. A read count matrix was defined as a data set for read count for next-generation sequencing, in which each row corresponds to a single gene and each column to a single cell. Samples in Cluster 1 were subjected to the cluster analysis and were divided into two subclusters, 1-a and 1-b. The difference between the expression level of a particular gene in each of these subclusters was statistically examined with an exact test. log_2_FC = log_2_ (average transcript level in subcluster 1-b) − log_2_ (average transcript level in subcluster 1-a) and log_2_CPM = {log_2_ (average transcript level in subcluster 1-a) + log_2_ (average transcript level in subcluster 1-b)}/2, where FC is the fold change and CPM is counts per million mapped reads. Differential expression analyses between other two neighboured clusters (e.g. Cluster 1 to 2) have been performed similarly (Supplementary Fig. 2b).

The kinetics of RNA levels for each gene along the virtual timeline has been deduced as follows. After plotting expression values [log_10_ (TPM + 0.01)] along the virtual timeline, sixth degree polynomial approximation was applied to the plots to draw the fitted curve (Fig. 4a–c; Supplementary Figs. 4c and 5a).

### Characterisation of candidate genes

For annotation of the detected genes, GO slim terms^35^ equipped in PomBase (https://www.pombase.org/)^36^ were adopted. To classify the 167 variable genes at the initial stage of germination, an enrichment analysis was applied (*p* < 0.05; Supplementary Fig. 3). Annotation and classification of variable genes between two neighbouring clusters have been performed similarly (Supplementary Fig. 2b).

The 167 variable genes were also classified according to their expression pattern along the virtual timeline (Supplementary Fig. 4). The expression profiles of the genes were subjected into the t-SNE analysis^37^ driven by the R package Rtsne ver. 0.15 (https://github.com/jkrijthe/Rtsne, 2015). Data of 64 single-cell based transcriptomes (64 dimensions) were used as an input, and the t-SNE operation reduced dimensions of the data set from 64 to 2. The DBSCAN algorithm^38^ included in the R package dbscan ver. 1.1.3 (https://CRAN.R-project.org/package=dbscan) was employed to identify clusters comprising genes with similar expression profiles. The graph (Supplementary Fig. 4b) was drawn with the R package factoextra ver. 1.0.5 (https://CRAN.R-project.org/package=factoextra).

### Microscopy

For live-cell imaging of GFP and differential interference-contrast microscopy, we applied our published standard methods^24^. Briefly, the observation system comprised the DeltaVision-SoftWoRx image acquisition system (Applied Precision) equipped with the Olympus inverted microscopes IX71 and IX81 and CoolSNAP HQ2 CCD cameras (Photometrics). In Fig. 4d–f and Supplementary Fig. 5b, the parental strain was induced for sporulation for 3 days at 26.5 °C, and spores were suspended with 200 µl ddH_2_O and mounted onto a glass-bottomed dish (Iwaki glass) precoated with lectin from Glycine max (Sigma). The dish was filled with 2 ml YE5S prior to observation. Spores that were seemingly ungerminated from its appearance^4^ were randomly chosen for live-cell imaging. Images were acquired every 5 min to measure any histone-GFP fluorescence (exposure time, 0.05 s) or every 15 min for other GFP fusion proteins (exposure time, 0.2 s). Images for 20 sections along the *Z* axis (0.2 µm interval) were filmed, deconvolved and finally projected to a single plane using the SoftWoRx software (v3.7.0 and v.6.5.1).

### Reporting summary

Further information on research design is available in the Nature Research Reporting Summary linked to this article.

## Supplementary information

Supplementary Information

Reporting Summary

## Data Availability

The data sets analysed during the current study have been deposited into NCBI Sequence Read Archive under the accession number PRJNA606890. The source data underlying Figs. 1d, 2b, 3b, 4a–c, g, i and 5b, c and Supplementary Figs. 3c, 4c, 5a, c, 6a, c, d and 7a, b are provided as a Source Data file.

## Source data

Source Data

## Code availability

The codes used in this study are available from the corresponding author on reasonable request.
