# Supplementary material for: Time-lapse single-cell transcriptomics reveals modulation of histone H3 for dormancy breaking in fission yeast
Source: Nat Commun. 2020 Mar 9;11:1265. doi: 10.1038/s41467-020-15060-y (PMC7062879; doi:10.1038/s41467-020-15060-y)
Supplement: Supplementary file 1 — Supplementary Information [file 41467_2020_15060_MOESM1_ESM.pdf]

Supplementary information

Time-lapse single-cell transcriptomics reveals modulation of histone H3 for dormancy breaking in fission yeast

Tsuyuzaki et al.

Supplementary Table 1. Strains used in this study

Strain	Alias	Genotype	Origin	Related figures
L975	Wild type	*h90*	Leupold, 1950	1c, 2a, 4g, 4h, 4i, 4j, S6d
HT0021	*ste11*-GFP (Wild type)	*h90 ste11-GFP-kan*	Our stock	1d, 1e, 1f, 2b, 2c, 2d
HT0059	Pnmt41-*bgs2*	*h90 bsd-Pnmt41-bgs2*	This study	2a, 2b, 2c, 2d, 3b, 3c, 3d, 3e, 4a
HT0254	H3.c1-GFP	*h90 hht1-GFP-kan*	This study	4d, 4k, 5b, S7a, S7b
HT0277	H3.c2-GFP	*h90 hht2-GFP-kan*	This study	4e, 5b, S7a, S7b
HT0278	H3.c3-GFP	*h90 hht3-GFP-kan*	This study	4f
HT0233	H3.c1KO	*h90 hht1::hph*	This study	4g, 4h, 4i, 4j, 5c, S6d
HT0242	H3.c2KO	*h90 hht2::bsd*	This study	4g, 4i, 4j
HT0252	H3.c3KO	*h90 hht3::nat*	This study	4g, 4i, 4j
HT0412	$P_{H3.c1}$→$P_{H3.c2}$	*h90 Phht1::ura4+::Phht2*	This study	5c
HT0435	$P_{H3.c1}$→$P_{H3.c2}$ H3.c1-GFP	*h90 Phht1::ura4+::Phht2 hht1-GFP-kan*	This study	5b, S7a, S7b
HT0338	$CDS_{H3.c1}$→$CDS_{H3.c2}$	*h90 hht1::ura4+::hht2*	This study	5c
HT0464	$CDS_{H3.c1}$→$CDS_{H3.c2}$-GFP	*h90 hht1::ura4+::hht2-GFP-kan*	This study	5b, S7a, S7b
HT0485	H3.c1→H3.c2	*h90 Phht1::Phht2 hht1::ura4+::hht2*	This study	5c
HT0489	H3.c1→H3.c2-GFP	*h90 Phht1::Phht2 hht1::ura4+::hht2-GFP-kan*	This study	5b, S7a, S7b
HT0752	*tim11*-GFP	*h90 tim11-GFP-kan*	This study	S5b, S5c
HT0754	*elo2*-GFP	*h90 elo2-GFP-kan*	This study	S5b, S5c
HT0761	*lys9*-GFP	*h90 lys9-GFP-kan*	This study	S5b, S5c
HT0765	*grs1*-GFP	*h90 grs1-GFP-kan*	This study	S5b, S5c
HT0759	*SPCC11E10.01*-GFP	*h90 SPCC11E10.01-GFP-kan*	This study	S5b, S5c
TM0010	h+ cen2-GFP	*h+ cen2-lacO-kan-ura4+ his7+<<lacI-NLS-GFP leu1 ura4 ade6*	Our stock	S6a
PZ809	h- cen2-GFP	*h- cen2-lacO-kan-ura4+ his7+<<GFP-lacI-NLS leu1 ade6*	Sakuno et al., 2009	S6a
HT0183	h+ H3.c1KO cen2-GFP	*h+ cen2-lacO-kan-ura4+ his7+<<lacI-NLS-GFP hht1::hph leu1 ura4 ade6*	This study	S6a

HT0192	h- H3.c1KO cen2-GFP	*h- cen2-lacO-kan-ura4+ his7+<<GFP-lacI-NLS hht1::hph leu1 ade6*	This study	S6a
HT0174	h- H3.c1KO	*h+ hht1::hph*	This study	S6b, S6c
HT0777	h+ H3.c1^{+}::*p*mo25-GFP Zfs1-mCherry::H3.c1	*h- hht1::pmo25-GFP-LK-kan zfs1-mCherry-hph::Z2::Phht1-hht1-Thht1-bsd*	This study	S6b, S6c

Supplementary Table 2. Oligonucleotides used in this study

oligo 1	5'-ATATGGATCCGGCGCGCCGTCGACT$_{(24)}$VN-3'
oligo 2	5'-ATATCTCGAGGGCGCGCCGGATCCT$_{(24)}$VN-3'
oligo 1_T15	5'-TATGGATCCGGCGCGCCGTCGACT$_{(15)}$-3'
V represents A, C, or G; N represents A, C, G, or T.	

Pnmt41-bgs2 oligo 1	5'-CGGTCTATTTTGCGTGTTTATTTATGTTTTG-3'
Pnmt41-bgs2 oligo 2	5'-GTTTAAACGAGCTCGAATTCCAGGTGTTTGTTTTCTATCTTCCTC TG-3'
Pnmt41-bgs2 oligo 3	5'-TATAGTCGCTTTGTTAAATCATGTCATGGCATGAACAAGATTACG-3'
Pnmt41-bgs2 oligo 4	5'-CGGTAATGGGGATCGAGTTTTC-3'
hht1 del oligo 1	5'-CGGATAGCAGGCTTAGTAATACCTTG-3'
hht1 del oligo 2	5'-TTAATTAACCCGGGGATCCGTATATCAAGCTAGTAGAATGAATA TC-3'
hht1 del oligo 3	5'-GTTTAAACGAGCTCGAATTCTTTTCTCCAGGACTTTTGGCAATTG-3'
hht1 del oligo 4	5'-GTGCAATTCTTTATGCGAACCGCC-3'
hht1-GFP oligo 1	5'-CTGCAGTACGCTTGCGTTTCC-3'
hht1-GFP oligo 2	5'-TTAATTAACCCGGGGATCCGTGAGCGTTCGCCACGGAG-3'
hht2 del oligo 1	5'-CACGAGTCTCTTCGTAAACCAAAGC-3'
hht2 del oligo 2	5'-TTAATTAACCCGGGGATCCGAACGTAATTCGGTAAATAAACTTAC AAAAAGG-3'
hht2 del oligo 3	5'-GTTTAAACGAGCTCGAATTCATGCATTGATTGCCTAATATTTTATT TGG-3'
hht2 del oligo 4	5'-CAGTAGCATCAATAATCCTGCAACAC-3'
hht2-GFP oligo 1	5'-CTGTGTAAGTGCTGTAGTTAGGCG-3'
hht2-GFP oligo 2	5'-TTAATTAACCCGGGGATCCGAGAGCGTTCGCCACGAAG-3'
hht3 del oligo 1	5'-GACGCTTCAAAGAGTAGACAACATCC-3'
hht3 del oligo 2	5'-TTAATTAACCCGGGGATCCGTATTTTCTAGTGTTCTGATAATTG CCCTG-3'
hht3 del oligo 3	5'-GTTTAAACGAGCTCGAATTCGCAACTTTACCAAATTGTGGTTGCC-3'
hht3 del oligo 4	5'-CCAAACTTGGAAAAACTCTTATGGGAC-3'
hht3-GFP oligo 1	5'-CTTTTCCACCTTTACCACGGCC-3'
hht3-GFP oligo 2	5'-TTAATTAACCCGGGGATCCGTGAGCGTTCACCGCGAAG-3'
tim11-GFP oligo 1	5'-GAATACGGTAAAGCGTTTGCTG-3'
tim11-GFP oligo 2	5'-TTAATTAACCCGGGGATCCGAACATTTTTTTCAAGTTCTTTGACAA ATTC-3'

tim11-GFP oligo 3	5’-GTTTAAACGAGCTCGAATTCATTCATCCTCTTGAACCACG-3’
tim11-GFP oligo 4	5’-GGAAGGCAATATACCTTTGATACC-3’
elo2-GFP oligo 1	5’-CGTTTTAGCTTGAATTGACAGATAC-3’
elo2-GFP oligo 2	5’-TTAATTAACCCGGGGATCCGTTTTTCATTGTTTAAACCTGAAGAAATAGG-3’
elo2-GFP oligo 3	5’-GTTTAAACGAGCTCGAATTCTGTTTTCGTGGTACCTCTTATGG-3’
elo2-GFP oligo 4	5’-CCAACCTTACACAACTGTAGTAC-3’
lys9-GFP oligo 1	5’-CCGAGTTTATTCATTGTTTGGTTGATATG-3’
lys9-GFP oligo 2	5’-TTAATTAACCCGGGGATCCGCTCTTCGTCAATAATTTCTTCCTCCAATC-3’
lys9-GFP oligo 3	5’-GTTTAAACGAGCTCGAATTCGCATGTTTCACGCTTTGGTTC-3’
lys9-GFP oligo 4	5’-GAAATTAGATGCATCAGAACAGGAAGC-3’
grs1-GFP oligo 1	5’-GCTCAACTTGTTGCTGAAGG-3’
grs1-GFP oligo 2	5’-TTAATTAACCCGGGGATCCGTTCTTGTGTAGACTTAAACTCTCC-3’
grs1-GFP oligo 3	5’-GTTTAAACGAGCTCGAATTCGTTACTTAACTCACTAGTATACTGGAAAG-3’
grs1-GFP oligo 4	5’-CAATTTGCTCCGGTGTATACTTC-3’
SPCC11E10.01-GFP oligo 1	5’-GATGCCTTATTGGTAATGGACAATAC-3’
SPCC11E10.01-GFP oligo 2	5’-TTAATTAACCCGGGGATCCGATTCTGTTTAAAATTTGCTTGAGCAAG-3’
SPCC11E10.01-GFP oligo 3	5’-GTTTAAACGAGCTCGAATTCTTTCGTCTATGAAGGCATAGTGG-3’
SPCC11E10.01-GFP oligo 4	5’-GAGTTTTCTTCCACCAACCAG-3’
zfs1-mCherry oligo 1	5’-GTTCATGGTCAACCGGAATC-3’
zfs1-mCherry oligo 2	5’-TTAATTAACCCGGGGATCCGAGGAGATTGCTTAATAGTTGCAC-3’
zfs1-mCherry oligo 3	5’-GTTTAAACGAGCTCGAATTCTATCACATCAGCTTGTATAAGATGAC-3’
zfs1-mCherry oligo 4	5’-GTATAGCCTGGTAGTTTTCTCG-3’

Supplementary figures

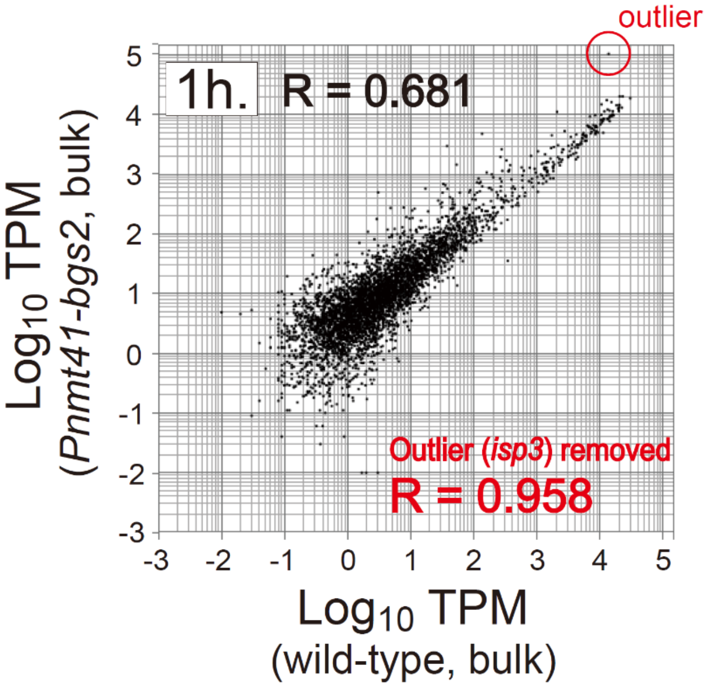

Supplementary Fig. 1

Comparison of transcriptomes derived from cultures of bulk spores.

This figure provides supplementary information for **Fig. 2d**, in which the correlation coefficient (R value) was 0.681 between transcriptomes from bulk WT and Pnmt41-*bgs2* cells. The red circle denotes the single outlier gene, *isp3*. The similarity between these two profiles increased (R = 0.958) when the data for *isp3* was not considered. We thus concluded that reduction of cellular Bgs2 in Pnmt41-*bgs2* cells did not affect the overall transcription profile except for that of *isp3*. Because Isp3 is a component of the surface layer of the spore wall (Fukunishi et al., Mol. Biol. Cell., 2014), a reduced level of Bgs2 may alter spore-wall integrity.

a

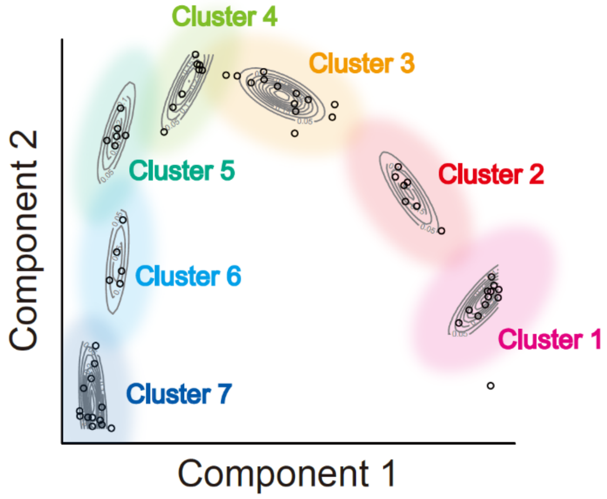

b

Transition	Differentially Expressed Genes	NOT Differentially Expressed Genes
Cluster 1 → Cluster 2	cell adhesion<br>establishment or maintenance of cell polarity<br>telomere organisation	cytoplasmic translation
Cluster 2 → Cluster 3	membrane organisation<br>cellular amino acid metabolic process<br>carbohydrate metabolic process<br>regulation of mitotic cell cycle phase transition<br>nucleobase-containing small molecule metabolic process	cytoplasmic translation<br>chromatin organisation
Cluster 3 → Cluster 4	generation of precursor metabolites and energy<br>nucleobase-containing small molecule metabolic process<br>cytoplasmic translation<br>apoptotic process<br>cellular amino acid metabolic process<br>lipid metabolic process	N/A
Cluster 4 → Cluster 5	generation of precursor metabolites and energy<br>cytoplasmic translation<br>protein folding<br>nucleobase-containing small molecule metabolic process<br>ribosome biogenesis<br>mRNA metabolic process<br>carbohydrate metabolic process<br>cellular amino acid metabolic process	vesicle-mediated transport<br>signaling
Cluster 5 → Cluster 6	nucleobase-containing small molecule metabolic process<br>generation of precursor metabolites and energy<br>carbohydrate metabolic process<br>membrane organisation<br>vesicle-mediated transport	regulation of transcription, DNA-templated
Cluster 6 → Cluster 7	carbohydrate metabolic process<br>generation of precursor metabolites and energy<br>nucleocytoplasmic transport<br>protein catabolic process<br>chromatin organisation	mitochondrial gene expression<br>cytoplasmic translation

Supplementary Fig. 2

Characterisation of germination processes using GO terms

a. The map of seven clusters of single-cell based transcriptomes (a reprise of **Fig. 3c**) along the virtual timeline covering the whole procedure of germination. **b.** Genes for which expression varied significantly between two clusters (e.g., Clusters 1 to 2) are selected, and GO slim terms that were significantly enriched ("Differentially Expressed Genes") and less enriched are shown ("NOT Differentially Expressed Genes"). Refer to **Supplementary Fig. 3** using similar methods for details.

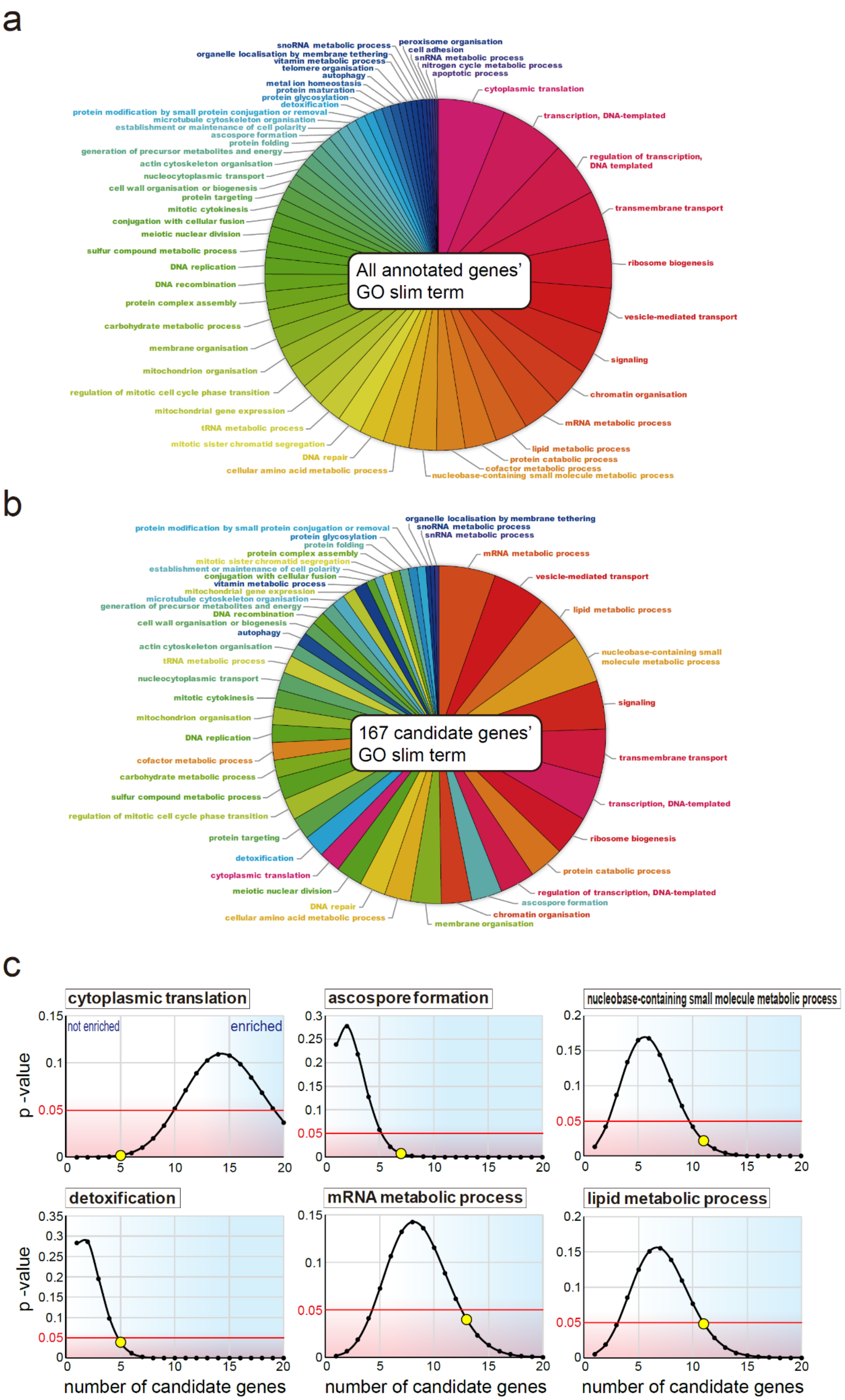

a
snoRNA metabolic process
organelle localisation by membrane tethering
vitamin metabolic process
telomere organisation
autophagy
metal ion homeostasis
protein maturation
protein glycosylation
detoxification
protein modification by small protein conjugation or removal
microtubule cytoskeleton organisation
establishment or maintenance of cell polarity
ascospore formation
protein folding
generation of precursor metabolites and energy
actin cytoskeleton organisation
nucleocytoplasmic transport
cell wall organisation or biogenesis
protein targeting
mitotic cytokinesis
conjugation with cellular fusion
meiotic nuclear division
sulfur compound metabolic process
DNA replication
DNA recombination
protein complex assembly
carbohydrate metabolic process
membrane organisation
mitochondrion organisation
regulation of mitotic cell cycle phase transition
mitochondrial gene expression
tRNA metabolic process
mitotic sister chromatid segregation
DNA repair
cellular amino acid metabolic process
peroxisome organisation
cell adhesion
snRNA metabolic process
nitrogen cycle metabolic process
apoptotic process
cytoplasmic translation
transcription, DNA-templated
regulation of transcription, DNA templated
transmembrane transport
ribosome biogenesis
vesicle-mediated transport
signaling
chromatin organisation
mRNA metabolic process
lipid metabolic process
protein catabolic process
cofactor metabolic process
nucleobase-containing small molecule metabolic process
All annotated genes' GO slim term
b
protein modification by small protein conjugation or removal
protein glycosylation
protein folding
protein complex assembly
mitotic sister chromatid segregation
establishment or maintenance of cell polarity
conjugation with cellular fusion
vitamin metabolic process
mitochondrial gene expression
microtubule cytoskeleton organisation
generation of precursor metabolites and energy
DNA recombination
cell wall organisation or biogenesis
autophagy
actin cytoskeleton organisation
tRNA metabolic process
nucleocytoplasmic transport
mitotic cytokinesis
mitochondrion organisation
DNA replication
cofactor metabolic process
carbohydrate metabolic process
sulfur compound metabolic process
regulation of mitotic cell cycle phase transition
protein targeting
detoxification
cytoplasmic translation
meiotic nuclear division
DNA repair
cellular amino acid metabolic process
organelle localisation by membrane tethering
snoRNA metabolic process
snRNA metabolic process
mRNA metabolic process
vesicle-mediated transport
lipid metabolic process
nucleobase-containing small molecule metabolic process
signaling
transmembrane transport
transcription, DNA-templated
ribosome biogenesis
protein catabolic process
regulation of transcription, DNA-templated
ascospore formation
chromatin organisation
membrane organisation
167 candidate genes' GO slim term
c
cytoplasmic translation
not enriched
enriched
ascospore formation
nucleobase-containing small molecule metabolic process
detoxification
mRNA metabolic process
lipid metabolic process
p -value
number of candidate genes
0
0.05
0.1
0.15
0.2
0.25
0.3
0.35
0
5
10
15
20

Supplementary Fig. 3

Characterisation of the variable 167 genes upon germination using GO terms

a. The proportion of gene ontology (GO slim) terms used to annotate all *S. pombe* genes is shown. GO slim terms are listed and coloured in order of frequency. **b.** GO slim terms for 167 genes variable in between subclusters 1-a and 1-b are shown. **c.** An enrichment analysis by hypergeometric test (two-sided, $p < 0.05$) identified six terms that were characteristic to the variable 167 genes (b) in comparison to all *S. pombe* genes (a). The first term “cytoplasmic translation” is significantly excluded (less enriched) in the 167 genes. Other five terms: “ascospore formation”, “nucleobase-containing small molecule metabolic process”, “detoxification”, “mRNA metabolic process” and “lipid metabolic process” are shown significantly enriched in the 167 genes. The Gaussian curve represent distribution of the number of candidate genes, the peak of which corresponds to the expected number of genes that have the indicated GO term among the 167 genes, calculated in proportion to the number of all genes. Yellow dots represent the actual number of genes with the GO slim term among the 167 genes. When the yellow dot is below the red lines ($p = 0.05$), the GO slim term is either enriched or excluded in the 167 genes. Source data are provided as a Source Data file.

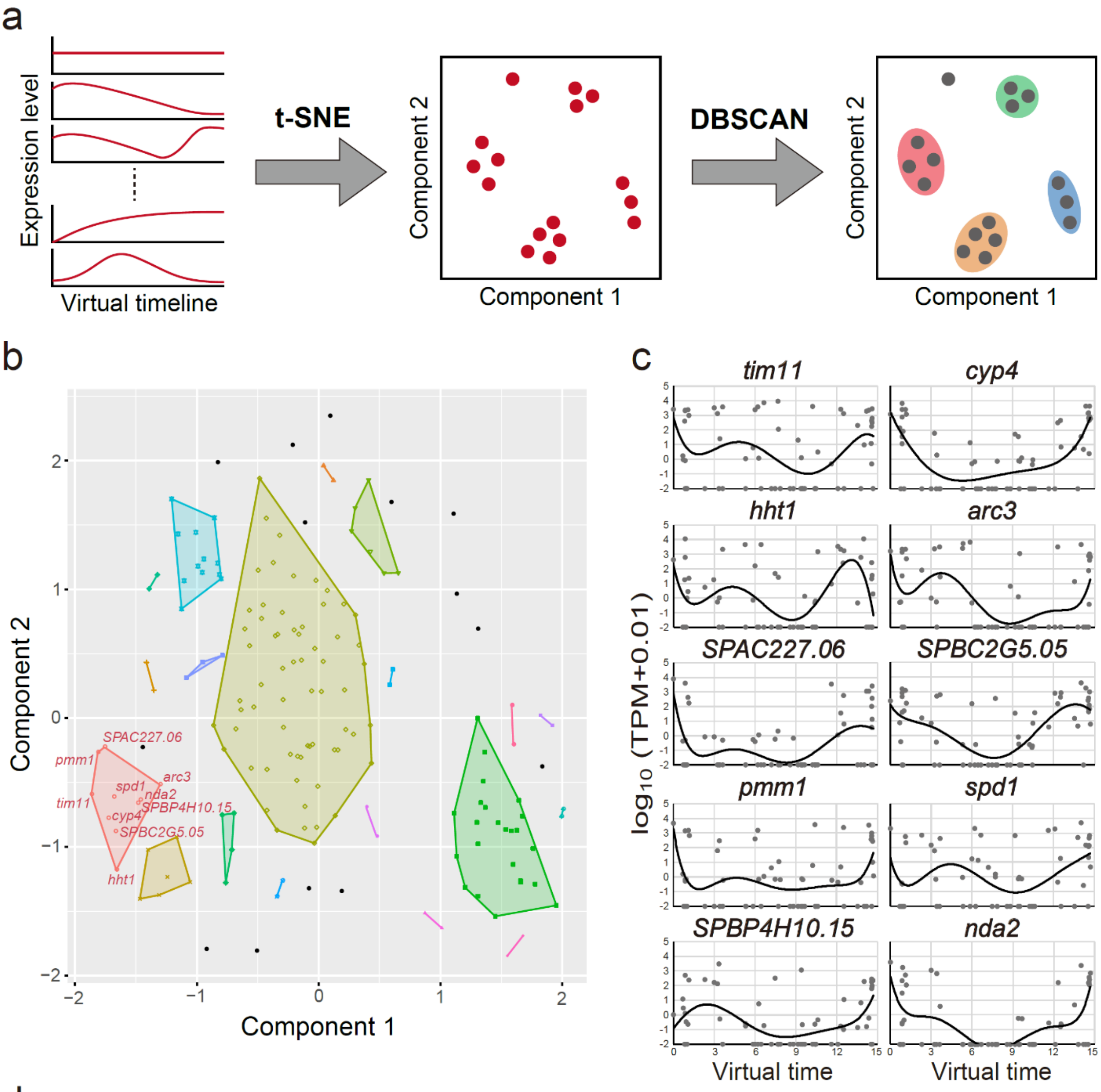

d

Gene name	GO slim 1	GO slim 2	GO slim 3	GO slim 4
tim11	nucleobase-containing small molecule metabolic process	transmembrane transport		
hht1	chromatin organisation			
SPAC227.06	vesicle-mediated transport			
pmm1	nucleobase-containing small molecule metabolic process			
SPBP4H10.15	mitochondrial gene expression	generation of precursor metabolites and energy	cellular amino acid metabolic process	
cyp4	protein folding			
arc3	vesicle-mediated transport	establishment or maintenance of cell polarity	actin cytoskeleton organisation	protein complex assembly
SPBC2G5.05	nucleobase-containing small molecule metabolic process	cofactor metabolic process		
spd1	nucleobase-containing small molecule metabolic process			
nda2	microtubule cytoskeleton organisation			

Supplementary Fig. 4

Classification of the variable 167 genes according to their expression patterns

a. The outline for classification of the 167 genes by their expression pattern during germination. The expression profiles of the 167 variable genes along the virtual timeline were subjected into the t-SNE analysis. Data of 64 single-cell based transcriptomes (meaning datasets comprising 64 dimensions) were used as an input, and the t-SNE operation reduced dimensions of the dataset from 64 to 2 (as illustrated with axes for Component 1 and 2). In the two-dimensional output plane, two plots locating closely to each other correspond to two genes that share similar expression profiles. To classify genes of similar expression patterns, DBSCAN was employed to identify clusters (as coloured in the right panel). **b.** The result of t-SNE and DBSCAN operations. Each plot represents either of the 167 genes. Each coloured cluster comprises genes of similar expression profiles. The red cluster contains 10 genes including the *hht1* (H3.c1) gene. **c.** Ten members that belongs to the red cluster are shown with their expression profile along the virtual timeline. All profiles show reduction of transcripts at the initial stage of germination, whereas increased later. **d.** GO slim terms annotated to the 10 members are listed. Four genes have the term “nucleobase-containing small molecule metabolic process”. Source data are provided as a Source Data file.

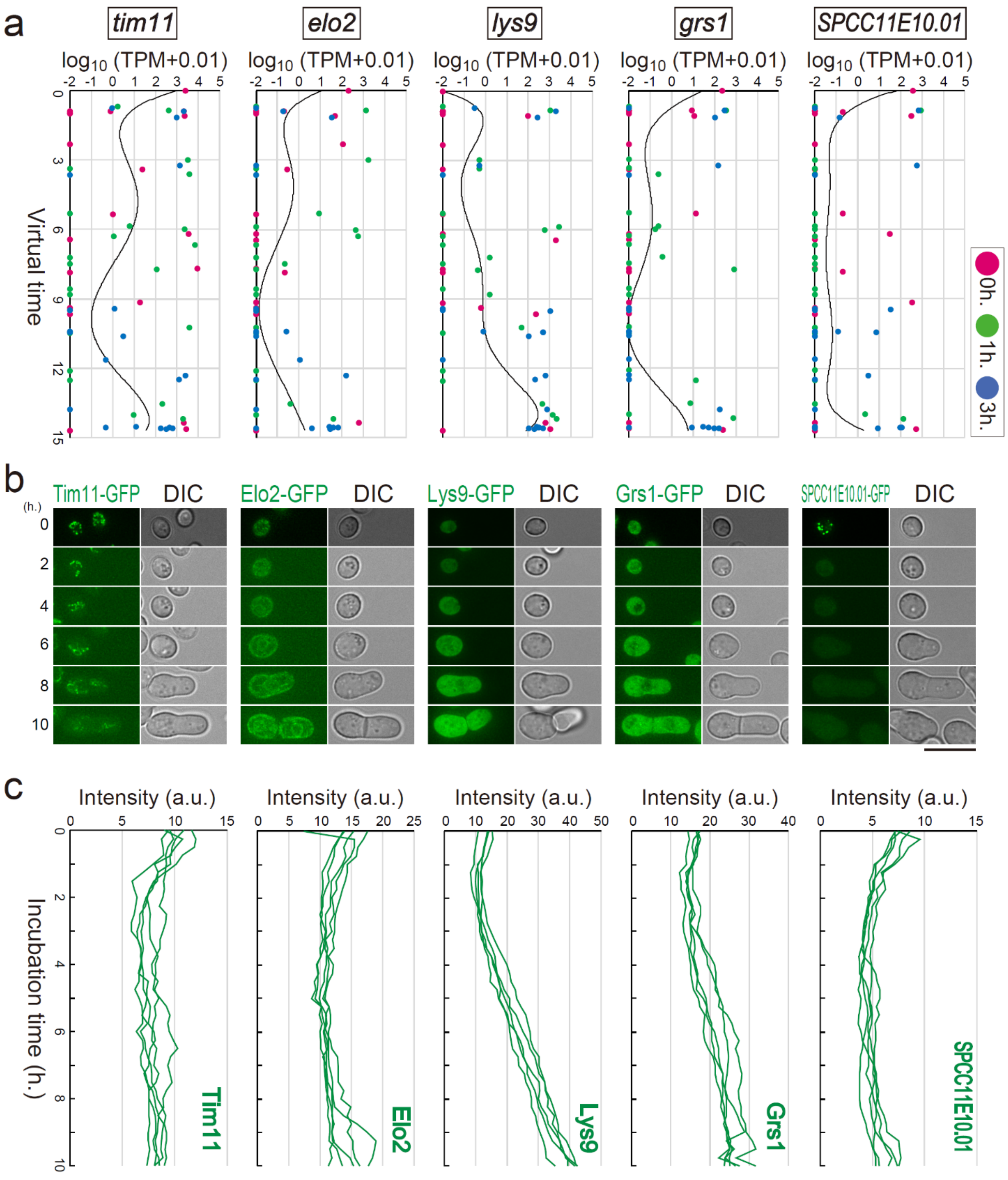

a
tim11
elo2
lys9
grs1
SPCC11E10.01
log10 (TPM+0.01)
Virtual time
0h.
1h.
3h.
b
Tim11-GFP
Elo2-GFP
Lys9-GFP
Grs1-GFP
SPCC11E10.01-GFP
DIC
(h.)
c
Intensity (a.u.)
Incubation time (h.)
Tim11
Elo2
Lys9
Grs1
SPCC11E10.01

Supplementary Fig. 5

Examples of other top genes in RNA and protein kinetics

a. Expression of other top variable genes detected in 64 transcriptomes shown in **Fig. 3f** besides *hht1* (top 3, **Fig. 4a**) was plotted versus the virtual timeline. Magenta, 0 h; green, 1 h; blue 3 h after feeding. RNA expression patterns of *tim11* (top 1), *elo2* (top 5), *lys9* (top 6), *grs1* (top 8) and *SPCC11E10.01* (top 9) are shown. **b, c.** Tim11-GFP (top 1), Elo2-GFP (top 5), Lys9-GFP (top 6), Grs1-GFP (top 8) and SPCC11E10.01-GFP (top 9) were expressed at the endogenous level under their native promoters. Live-cell imaging started from dormant spores (b), and GFP fluorescence intensity was quantified (c). The whole-cell florescence intensity of GFP was quantified along the time. Data for 5 individual cells are shown. Other four top genes are not applicable for the assay: *meu19* (top 2) is a non-coding RNA, and transcript levels of *tim9* (top 4), *spo6* (top 7) and *ppa2* (top 10) did not alter over time. Data are representative of >5 independent cells. Scale bar, 10 μm. Source data are provided as a Source Data file.

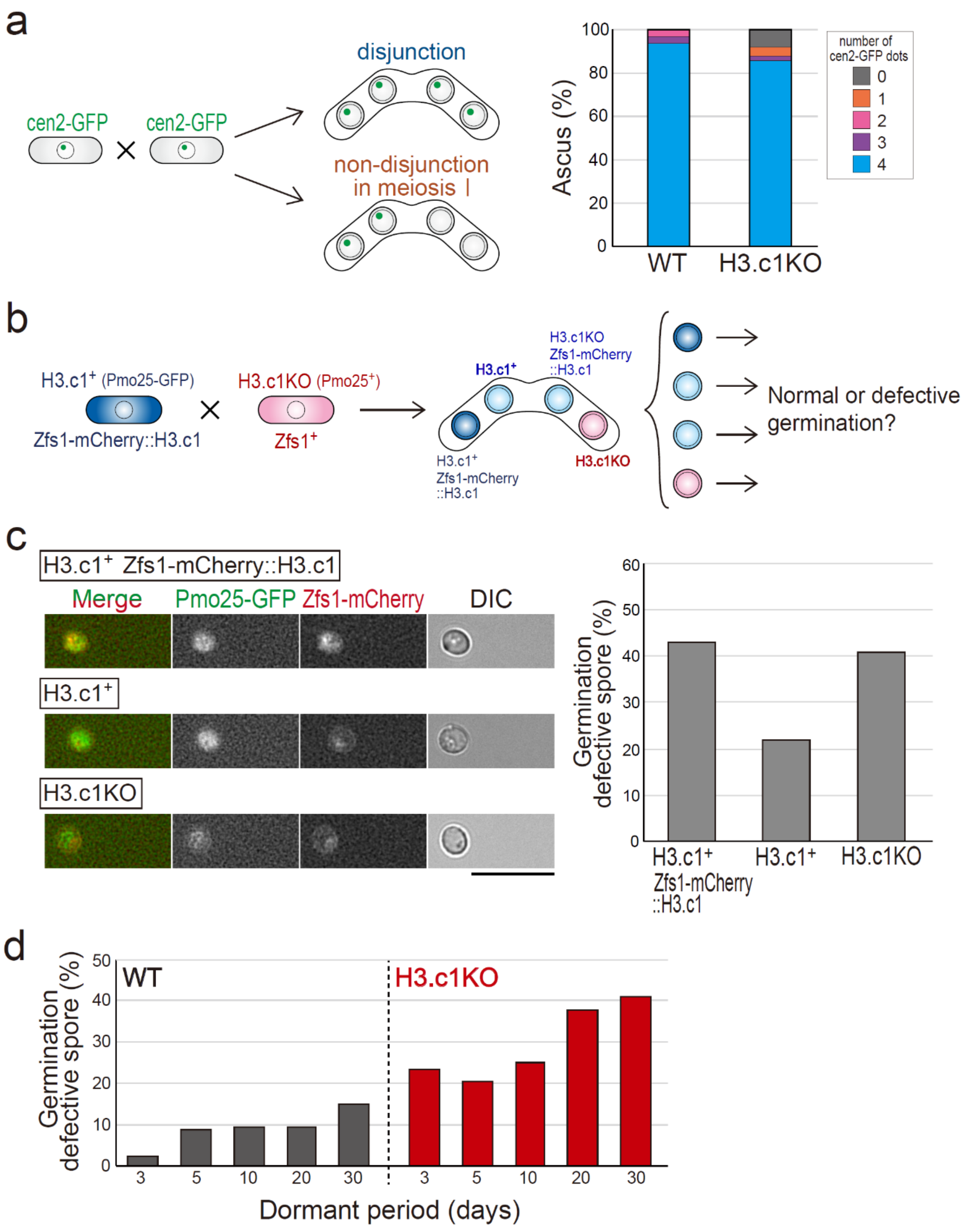

a
cen2-GFP
cen2-GFP
disjunction
non-disjunction in meiosis I
100
80
60
40
20
0
Ascus (%)
WT
H3.c1KO
number of cen2-GFP dots
0
1
2
3
4
b
H3.c1+ (Pmo25-GFP)
Zfs1-mCherry::H3.c1
H3.c1KO (Pmo25+)
Zfs1+
H3.c1+
H3.c1KO Zfs1-mCherry ::H3.c1
H3.c1+ Zfs1-mCherry ::H3.c1
H3.c1KO
Normal or defective germination?
c
H3.c1+ Zfs1-mCherry::H3.c1
Merge
Pmo25-GFP
Zfs1-mCherry
DIC
H3.c1+
H3.c1KO
Germination defective spore (%)
60
50
40
30
20
10
0
H3.c1+ Zfs1-mCherry ::H3.c1
H3.c1+
H3.c1KO
d
WT
H3.c1KO
Germination defective spore (%)
50
40
30
20
10
0
3
5
10
20
30
3
5
10
20
30
Dormant period (days)

Supplementary Fig. 6

Meiosis, sporulation and spore maintenance are not defective in H3.c1KO cells

a. Chromosome segregation pattern during meiosis of WT and H3.c1KO zygotes. Both spores showed 4 dots of cen2-GFP in an ascus (1 : 1 : 1 : 1), indicating that two consecutive rounds of meiotic chromosome segregation were normal (disjunction). If non-disjunction of homologous chromosomes occurs at meiosis I, for instance, cen2-GFP pattern after meiosis becomes 1 : 1 : 0 : 0 in an ascus (non-disjunction). $n > 30$ asci for each strain were examined (N = 1 experiment). **b.** A schematic for "littermate" experiments, designed to compare H3.c1^{+} and H3.c1KO spores generated from same parents. H3.c1^{+} and H3.c1KO cells were conjugated and zygotic meiosis was induced. One of the parental strains (H3.c1^{+}) has been engineered in two respects as follows: (1) H3.c1^{+} gene is linked to the Pmo25-GFP gene, which enables identification of genotypes (H3.c1^{+} or H3.c1KO) in offspring spores. (2) If H3.c1KO and H3.c1^{+} cells were used as parents, the resulting diploid zygote would possess only a single copy of H3.c1^{+} gene, which might cause defects during meiosis because of possible haploinsufficiency. To avoid this concern, an additional H3.c1^{+} gene was inserted into a chromosome of the H3.c1^{+} parent strain. The H3.c1^{+} gene including the native promoter and terminator was inserted next to the *zfs1-mcherry* locus, referred to as "Zfs1-mCherry::H3.c1". When germination is induced, offspring spores of our interest should not contain the additional H3.c1^{+} gene, as it results in overexpression. For that purpose, we compared germination of spores showing GFP(+) mCherry(–) (meaning H3.c1^{+} without the additional H3.c1^{+} gene) and GFP(–) mCherry(–) (H3.c1KO without the additional gene). **c.** "Littermate" spores were tested for germination. As germination of the H3.c1KO [GFP(–) mCherry(–)] spores were twice more defective than the H3.c1^{+} [GFP(+) mCherry(–)] spores. This indicates that the defects seen in H3.c1KO spores in germination are not originated from meiosis and sporulation of parental cells. As a reference, images and a graph for H3.c1^{+} spores with the additional H3.c1^{+} gene [GFP(+) mCherry(+)] are shown. Pmo25-GFP expression in zygotes caused some meiotic/sporulation defects, which resulted in germination defects even in H3.c1^{+} spores. $n > 100$ spores, N = 2 independent experiments. Values averaged in the graph are given below. H3.c1^{+} Zfs1-mCherry::H3.c1, 35.9% and 50.0%; H3.c1^{+}, 22.6% and 21.4%; H3.c1KO, 36.8% and 44.7%. Scale bar, 10 µm. **d.** Assays to test maintenance of the spore integrity during dormancy. After generation of WT and H3.c1KO spores, those were stored in dormant conditions for indicated days (3–30 days), and germination was induced. Percentages of cells that showed germination defects are shown. $n > 100$ spores for each strain were examined (N = 1 experiment). Source data are provided as a Source Data file.

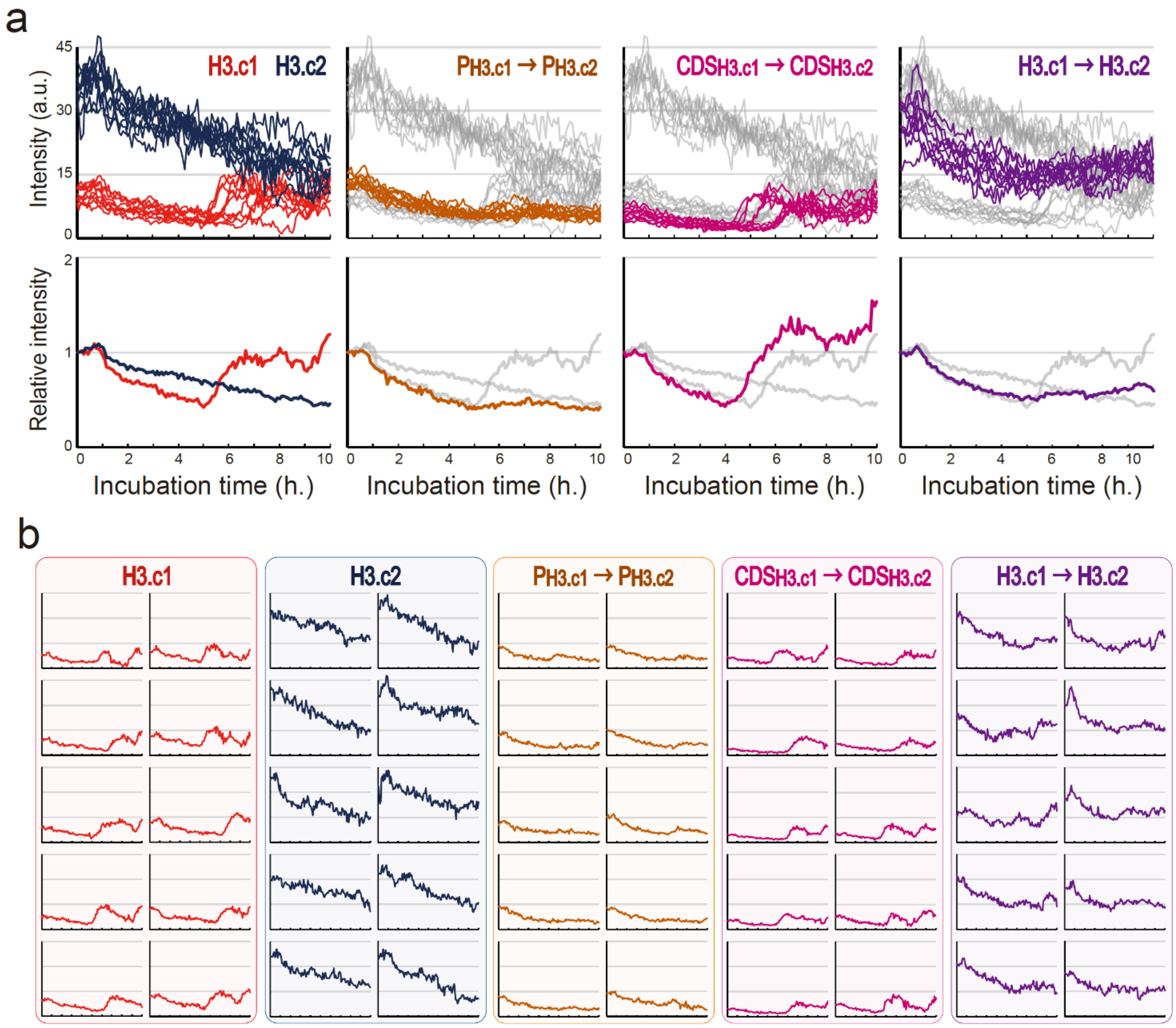

Supplementary Fig. 7

Examples of H3-GFP fluorescence kinetics along the real timeline

Kinetics of fluorescence intensity of five histone H3-GFP constructs in representative 10 cells is shown. Five representative examples therefrom are chosen and shown in **Fig. 5b** for simplicity. The data of 10 samples are shown overlapped in a single layer (a) and separately (b). Source data are provided as a Source Data file.

Figure 1d

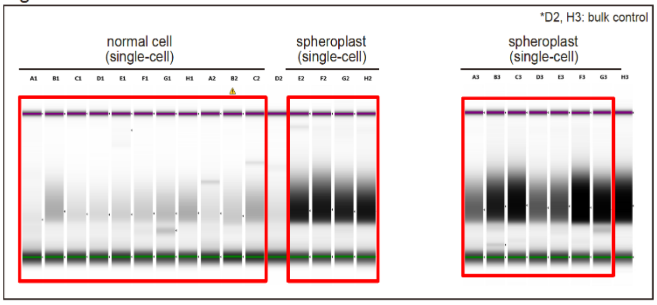

Figure 2b

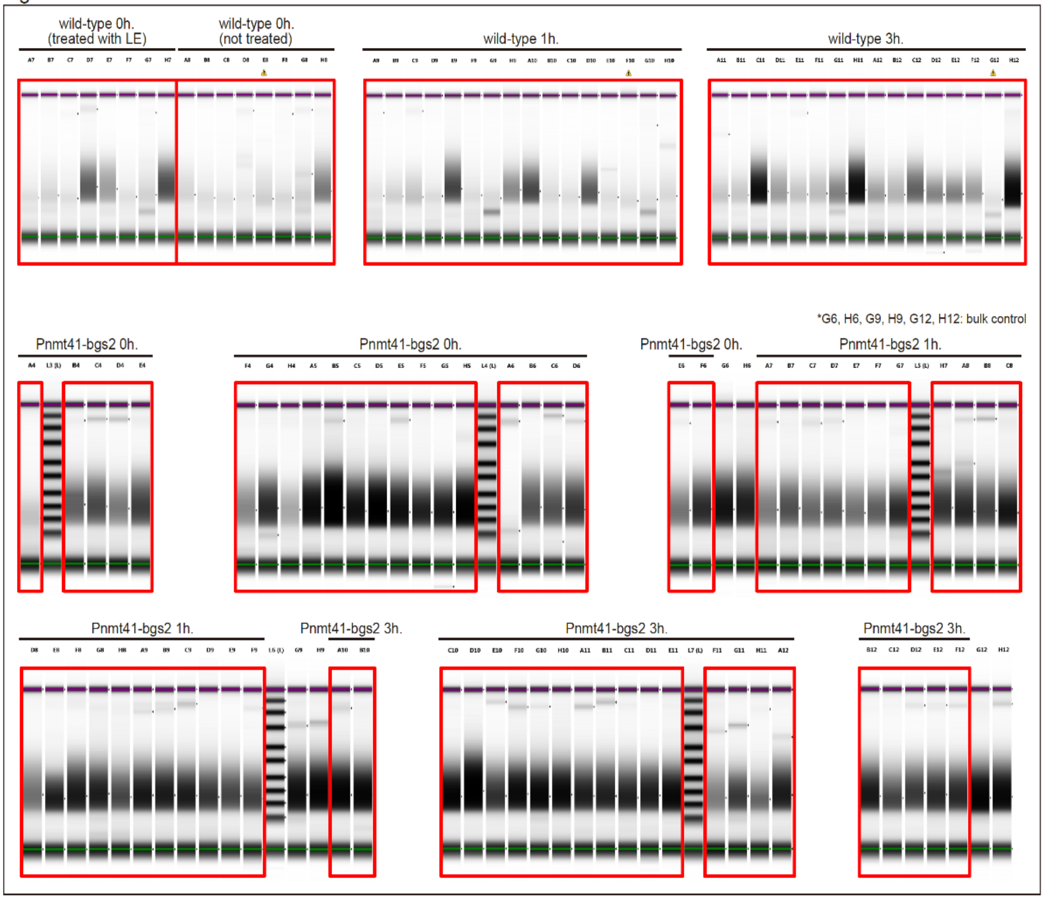

Figure 4j

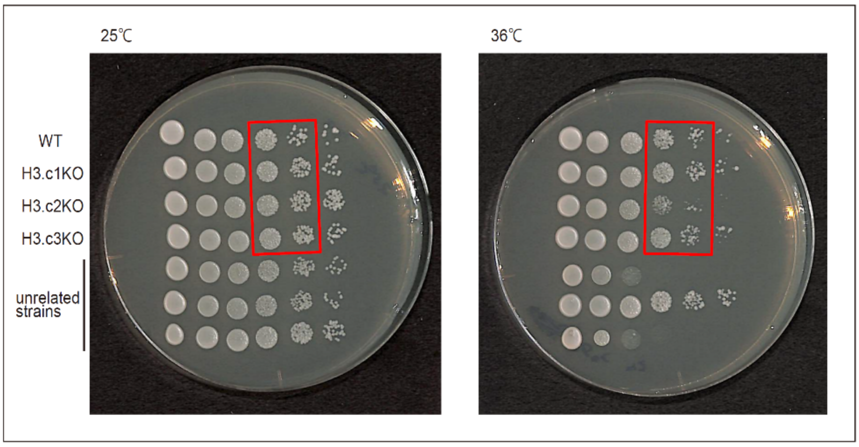

Supplementary Fig. 8

Uncropped original images for electrophoresis and plates
