# Supplementary material for: Time-lapse single-cell transcriptomics reveals modulation of histone H3 for dormancy breaking in fission yeast
Source: Nat Commun. 2020 Mar 9;11:1265. doi: 10.1038/s41467-020-15060-y (PMC7062879; doi:10.1038/s41467-020-15060-y)
Supplement: Supplementary file 2 — Reporting Summary [file 41467_2020_15060_MOESM2_ESM.pdf]

nature research

Corresponding author(s): MASAMITSU SATO

Last updated by author(s): Feb 16, 2020

Reporting Summary

Nature Research wishes to improve the reproducibility of the work that we publish. This form provides structure for consistency and transparency in reporting. For further information on Nature Research policies, see Authors & Referees and the Editorial Policy Checklist.

Statistics

For all statistical analyses, confirm that the following items are present in the figure legend, table legend, main text, or Methods section.

n/a	Confirmed	
☐	☒	The exact sample size (*n*) for each experimental group/condition, given as a discrete number and unit of measurement
☐	☒	A statement on whether measurements were taken from distinct samples or whether the same sample was measured repeatedly
☐	☒	The statistical test(s) used AND whether they are one- or two-sided<br>*Only common tests should be described solely by name; describe more complex techniques in the Methods section.*
☒	☐	A description of all covariates tested
☒	☐	A description of any assumptions or corrections, such as tests of normality and adjustment for multiple comparisons
☒	☐	A full description of the statistical parameters including central tendency (e.g. means) or other basic estimates (e.g. regression coefficient) AND variation (e.g. standard deviation) or associated estimates of uncertainty (e.g. confidence intervals)
☐	☒	For null hypothesis testing, the test statistic (e.g. *F*, *t*, *r*) with confidence intervals, effect sizes, degrees of freedom and *P* value noted<br>*Give P values as exact values whenever suitable.*
☒	☐	For Bayesian analysis, information on the choice of priors and Markov chain Monte Carlo settings
☒	☐	For hierarchical and complex designs, identification of the appropriate level for tests and full reporting of outcomes
☒	☐	Estimates of effect sizes (e.g. Cohen's *d*, Pearson's *r*), indicating how they were calculated

Our web collection on statistics for biologists contains articles on many of the points above.

Software and code

Policy information about availability of computer code

Data collection	SoftWoRx v3.7.0 and v6.5.1 (Applied Precision) for image acquisition in microscopy
Data analysis	scRNA-seq data were analysed using open source code RSEM v1.3.0, monocle v2.4.0, mclust v5.3, amap v0.8.14, dendextend v1.8.0, edgeR v3.18.1, Rtsne v0.15, dbscan v1.1.3, factoextra v1.0.5, software R v3.4.1 and Subio Platform v1.19, as described in the method section of the manuscript.

For manuscripts utilizing custom algorithms or software that are central to the research but not yet described in published literature, software must be made available to editors/reviewers. We strongly encourage code deposition in a community repository (e.g. GitHub). See the Nature Research guidelines for submitting code & software for further information.

Data

Policy information about availability of data

All manuscripts must include a data availability statement. This statement should provide the following information, where applicable:

- Accession codes, unique identifiers, or web links for publicly available datasets
- A list of figures that have associated raw data
- A description of any restrictions on data availability

scRNA-seq data analysed during the current study have been deposited into NCBI Sequence Read Archive (accession number: PRJNA606890). Schizosaccharomyces_pombe.ASM294v2.25. is referenced in RefSeq (accession number: GCF_000002945.1). S. pombe bulk RNA-seq data (Marguerat et al., 2012) is referenced in ArrayExpress (accession number: E-MTAB-1154).

Field-specific reporting

Please select the one below that is the best fit for your research. If you are not sure, read the appropriate sections before making your selection.

☒ Life sciences ☐ Behavioural & social sciences ☐ Ecological, evolutionary & environmental sciences

For a reference copy of the document with all sections, see nature.com/documents/nr-reporting-summary-flat.pdf

Life sciences study design

All studies must disclose on these points even when the disclosure is negative.

Sample size	For scRNA-seq analysis, total 77 samples (n = 11 for vegetative cells, n = 66 for spore cells) as disclosed in the main text.<br>n = 11 vegetative cells were sufficient because of its correlation with bulk sample, as described in the main text.<br>n = 66 spores were sufficient, because results with a similar tendency could be obtained with a reduced sample size (data not shown).
Data exclusions	Quality control test was performed prior to scRNA-seq analysis to exclude samples that did not yield any cDNA amplification, as menthioned in this text. Filtering for monocle analysis was performed to exclude low coverage RNA-seq data.
Replication	For spheroplasting assay, there were N>10 biologically independent experiments. For scRNA-seq, N=2 independent experiments using vegetative cells were done. Having confirmed validity (reproducibility) of those methods, an RNA-seq experiment of spore germination was done.<br>For Pnmt41-bgs2 sporulation assay, there were n=3 biologically independent experiments.<br>For the time-lapse DIC microscopy for germination, N=2 independent experiments were performed for each strain to confirm reproducibility.<br>For the other time-lapse observation for GFP fluorescence, one experiment was done and n > 10 cells were observed through time-lapse imaging to show average fluorescence intensities.
Randomization	Single cells were randomly chosen and picked up for further scRNA-seq studies.<br>For microscopy observation and quantification, cells were randomly chosen.
Blinding	All analyses were monitored by at least two people.

Reporting for specific materials, systems and methods

We require information from authors about some types of materials, experimental systems and methods used in many studies. Here, indicate whether each material, system or method listed is relevant to your study. If you are not sure if a list item applies to your research, read the appropriate section before selecting a response.

Materials & experimental systems

n/a	Involved in the study	
☒	☐	Antibodies
☒	☐	Eukaryotic cell lines
☒	☐	Palaeontology
☒	☐	Animals and other organisms
☒	☐	Human research participants
☒	☐	Clinical data

Methods

n/a	Involved in the study	
☒	☐	ChIP-seq
☒	☐	Flow cytometry
☒	☐	MRI-based neuroimaging
